# Variation in pentose phosphate pathway-associated metabolism dictates cytotoxicity outcomes determined by tetrazolium reduction assays

**DOI:** 10.1038/s41598-023-35310-5

**Published:** 2023-05-22

**Authors:** Jayme P. Coyle, Caroline Johnson, Jake Jensen, Mariana Farcas, Raymond Derk, Todd A. Stueckle, Tiffany G. Kornberg, Yon Rojanasakul, Liying W. Rojanasakul

**Affiliations:** 1 HELD/ACIB, National Institute for Occupational Safety and Health, Morgantown, WV USA; 2 Department of Environmental Health, Harvard University, Boston, MA USA; 3 Department of Pharmaceutical Sciences, West Virginia University, Morgantown, WV USA; 4 Present Address: Centers for Disease Control and Prevention, National Institute for Occupational Safety and Health, 1095 Willowdale Rd., Morgantown, WV 26505 USA

**Keywords:** Biochemistry, Biological techniques, Cell biology

## Abstract

Tetrazolium reduction and resazurin assays are the mainstay of routine in vitro toxicity batteries. However, potentially erroneous characterization of cytotoxicity and cell proliferation can arise if verification of baseline interaction of test article with method employed is neglected. The current investigation aimed to demonstrate how interpretation of results from several standard cytotoxicity and proliferation assays vary in dependence on contributions from the pentose phosphate pathway (PPP). Non-tumorigenic Beas-2B cells were treated with graded concentrations of benzo[a]pyrene (B[a]P) for 24 and 48 h prior to cytotoxicity and proliferation assessment with commonly used MTT, MTS, WST1, and Alamar Blue assays. B[a]P caused enhanced metabolism of each dye assessed despite reductions in mitochondrial membrane potential and was reversed by 6-aminonicotinamide (6AN)—a glucose-6-phosphate dehydrogenase inhibitor. These results demonstrate differential sensitivity of standard cytotoxicity assessments on the PPP, thus (1) decoupling “mitochondrial activity” as an interpretation of cellular formazan and Alamar Blue metabolism, and (2) demonstrating the implicit requirement for investigators to sufficiently verify interaction of these methods in routine cytotoxicity and proliferation characterization. The nuances of method-specific extramitochondrial metabolism must be scrutinized to properly qualify specific endpoints employed, particularly under the circumstances of metabolic reprogramming.

## Introduction

Tetrazolium reduction assays are widely employed screening techniques for the measurement of proliferation and cytotoxicity in vitro within academic and industry settings. Their relative ease of use, simplistic quantitative detection modality, and broad dynamic range offer advantages over traditional proliferation assessment methods, some of which require luminescent detection or radiolabeling. Mosmann^1^ correlated 3-(4,5-dimethylthiazol-2-yl)-2,5-diphenyltetrazolium bromide (MTT) reduction to cell number and validated the method as an indicator of mitogen-induced proliferation. Therefore, under basal metabolic conditions MTT reduction can serve as a reliable proxy of proliferation.

Briefly after the development of MTT, water-soluble cytotoxicity and proliferation reagents allowed for adaptation and application into high-throughput screening platforms. Many of these novel reagents fall within the water soluble tetrazolium salt family, e.g., water soluble tetrazolium 1 and 8, WST1/8,^2,3^, MTS (3-[4,5,dimethylthiazol-2-yl]-5-[3-carboxymethoxy-phenyl]-2-[4-sulfophenyl]-2H-tetrazolium)^4^, and XTT (2,3-bis-(2-methoxy-4-nitro-5-sulfophenyl)-2H-tetrazolium-5-carboxanilide)^5^, which require electron coupling agents to transfer reduction equivalents in order to metabolize extracellularly-localized tetrazolium salts^6^. The presumptive mechanism of tetrazolium metabolism among applied sciences remained focused upon mitochondrial dehydrogenase activity as the source of reduction equivalents^7–9^, despite indications of extramitochondrial MTT metabolism in localization and fractionation studies^6,10–13^. In addition to water-soluble tetrazolium salts, fluorescently active resazurin analogues have also been developed and implemented for routine cytotoxicity/proliferation screening^14^. Fractionation studies by Gonzalez and Tarloff^11^ confirmed cytosolic and microsomal metabolism of resazurin, in addition to mitochondrial metabolism, thereby uncoupling metabolism of resazurin specifically to a mitochondrial origin.

Metabolism of tetrazolium salt methods described depend, in part, on cofactors other than NADH, e.g., NAPDH, glutathione, pyruvate, etc.^15^. Detailed investigations into ascribing cofactor-specific metabolism within metabolically competent cells have not yet been undertaken. Studies employing pharmacological inhibitors are beginning to attribute tetrazolium reduction with functional intracellular systems, e.g., glycolysis, Citric Acid Cycle (TCA), Pentose Phosphate Pathway (PPP), etc. Stepanenko and Dmitrenko^16^ demonstrated MTT reduction relied upon additional mechanisms besides alterations in mitochondrial dehydrogenase metabolic status, potentially leading to incorrect interpretations of cytotoxicity and proliferative capacity in vitro. These findings were consistent with contemporaneous findings^17,18^. Xie, et al.^19^ attempted to elucidate glycolytic influence of intracellular WST8 metabolism using a battery of pharmacological inhibitors, though the authors failed to account for cytotoxicity of their key inhibitor: iodoacetate^20^. Kelly, et al. ^21^ bridged that knowledge by demonstrating primacy of WST1 metabolism on functional glucose-6-phosphate dehydrogenase (G6PDH) using dehydroepiandrosterone—a known G6PDH inhibitor. Dehydroepiandrosterone significantly reduced basal WST1 metabolism but did not affect the downstream enzymatic steps of glycolysis. This indirectly implicated the PPP as the primary, but not exclusive, source of cytosolic-derived labile reduced nicotinamide adenine dinucleotide phosphate (NADPH)^22–24^. Similar studies examining differential contribution of PPP-derived NADPH to resazurin and other tetrazolium salts are lacking.

Aside from generating nucleotide and amino acids to support proliferation^25^, kinetic changes in the PPP to sustain NADPH:oxidoreductases are required for sustaining microsomal metabolism^26,27^. Benzo[a]pyrene (B[a]P), a ubiquitous environmental carcinogen, induces robust cytochrome P450 A1/B1 (CYP1A1/B1) transcription primarily through ligation with and nuclear translocation of the Aryl Hydrocarbon Receptor (AhR) in conjunction with AhR nuclear translocator to the Xenobiotic Response Element sequence in mammalian cells^28,29^. This leads to transcriptional up-regulation of phase I and phase II enzymes to increase capacity for B[a]P metabolism and clearance^30^. To support continued CYP1A/B1 metabolism, NADPH:P450 oxidoreductases, responsible for regenerating the active site of CYP450 isoforms, require a steady supply of cytosolic NADPH^31^. Though a substantial portion of cytosolic NADPH is derived from the PPP, non-PPP sources, such as isocitrate dehydrogenase-1 and malic enzyme-1^22–24^, also contribute to the cytosolic pool to fuel microsomal metabolism. Consequently, changes in NADPH could lead to erroneous interpretation of tetrazolium or resazurin methods for those which significantly depend on this cofactor for metabolism. Under such conditions, changes in tetrazolium or resazurin metabolism due to test articles must be placed within the context of confounding sources of metabolism to faithfully correlate endpoints with proliferation and cytotoxicity^32^. Furthermore, reconciliation of inherently differential metabolic profiles of each colorimetric/fluorometric endpoint must be realized within each tissue culture model. Failure to do under circumstances of de-coupled endpoint metabolism from proliferative capacity could potentially lead to erroneous interpretation.

The current investigation aimed to associate comparative sensitivity of routine colorimetric and fluorometric cytotoxicity/proliferation reporters in Beas-2B cells to PPP metabolism. The model system was chosen given the continued emphasis and research in polycyclic aromatic hydrocarbon (PAH) carcinogenesis in human lung epithelia. While several differing in vitro models are currently employed, the human bronchial epithelial cell culture, Beas-2B, remains a widely accepted non-carcinoma model for test agents induced pulmonary epithelial cell responses. However, the interaction between PAH-induced metabolic changes and protochromo-/fluorophore metabolism of widely used tetrazolium and resazurin agents used for cytotoxicity/proliferation testing in the Beas-2B model has not been investigated to exclude confounding, particularly arising from mobilization of intracellular NADPH production via the PPP. Therefore, the objective of present study is to exploit the PAH-Beas-2B model to determine and compare sources of metabolism by several such routine cytotoxicity/proliferation methods to understand underlying biochemical-metabolic mechanisms and meanings of the observed endpoint.

## Results

### Beas-2B proliferative capacity

**Figure 1:**
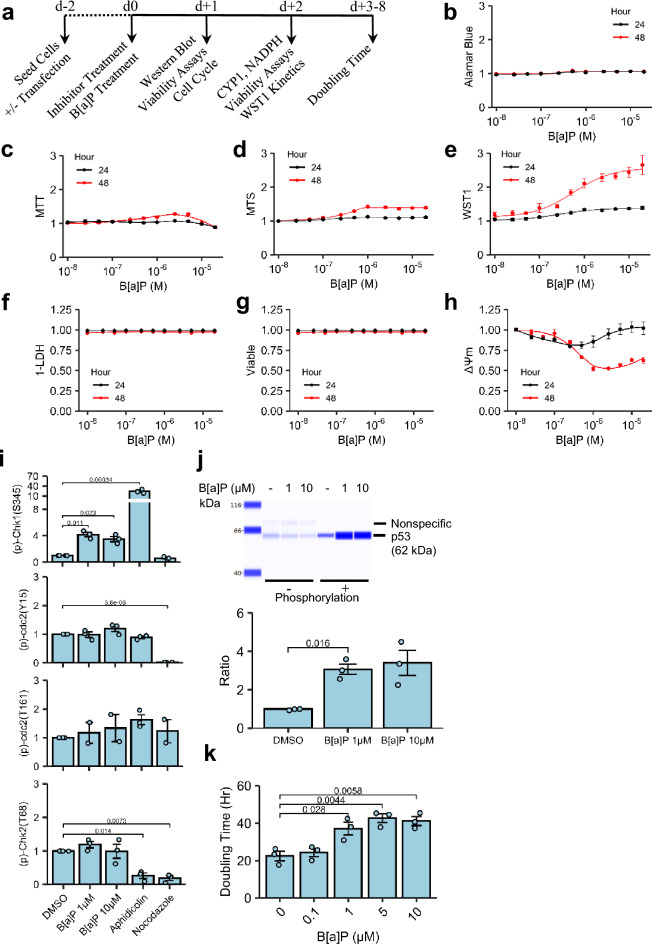
B[a]P-Induced Metabolic and Proliferative Changes. (**a**) Treatment Paradigm. (**b–h**) Beas-2B cells treated with graded concentrations B[a]P were assessed with resazurin/tetrazolium salts of interest: (**b**) Alamar Blue, (**c**) MTT, (**d**) MTS, (**e**) WST1. Beas-2B cells were assessed for membrane leakage via (**f**) LDH release and (**g**) live-cell imaging propidium iodide uptake as well as ΔΨm via (**h**) JC-1 ratiometric analysis. All endpoints were assessed at 24 (Black Circles) and 48 Hours (Red Circles). n = 3. (**i**) Beas-2B cells treated with B[a]P for 24 h, followed by semi-quantitative Western analysis of cell cycle regulators using total protein staining normalization as the loading control; 3 µg/mL aphidicolin (18 h) and 100 ng/mL nocodazole (18 h) were included for G1/S and G2/M controls, respectively. n = 3, except for (p)-cdc2(T161) assessment of B[a]P- and nocodazole-treated Beas-2B cells for which n = 2. Full-length capillary electrophoresis images are presented in Supplementary Fig. 1(p)-Chk1(S345), (p)-Chk2(T68), (p)-cdc2(Y15), and total protein normalization loading controls, Supplementary Fig. 2 (p)-cdc2(T161) and total protein normalization loading control for controls and B[a]P, Supplementary Fig. 3 (p)-cdc2(T161) and total protein normalization loading control for aphidicolin and nocodazole. Supplementary Fig. 10 provides the Western Blot images. (**j**) Similar to cell cycle regulators, p53 activation via phosphorylation at serine 15 was quantitated as a ratio of (p)-p53(S15) and p53. Though not significant, the significance test between controls and the 10 µM B[a]P is shown for comparison. n = 3. Full-length capillary electrophoresis is presented in Supplementary Fig. 4 For display, the presented representative images were grouped in the Compass software. (**k**) Beas-2B cells were treated with graded concentrations of B[a]P for 3 days, followed by doubling time assessment using WST. n = 3. Endpoints are the arithmetic mean listed independent experiments with error bars denoting one standard error of the mean. Small variance of estimates in panels B-H may not be too small to be visualized.

Following treatment of Beas-2B cells with B[a]P, metabolic assessments using four different cytotoxicity/proliferation assays (WST1, MTS, MTT, and Alamar Blue) resulted in four distinct metabolic profiles, with substantial hyperstimulatory B[a]P-dependent metabolism. When assessed by Alamar Blue, stimulatory metabolism did not exceed 10% above untreated controls at any dose tested. Neither MTS nor MTT induced appreciable hyperstimulatory metabolism 24 h-post treatment, while hpyerstimulatory metabolism was observed with at least one dose at 48 h.. WST1 showed appreciable hyperstimulatory metabolism 24 h post-treatment that was accentuated 48 h post-treatment (Fig. 1b–e). For all of the methods tested, with the exception of MTT, B[a]P-treated cells reached a plateau up to the highest concentration of 20 µM. MTT first peaked at 127.8% ± 1.8% (p < 0.001, 2.5 µM B[a]P treatment), but deflected towards control levels at higher concentrations (Fig. 1c). Based on measurements at 48 h, B[a]P induced hyperstimulatory metabolism of colorimetric/fluorometric endpoints in the following ordered rank: WST1 > MTS > MTT > Alamar Blue.

Confirmatory measures of potential cytotoxicity were employed to characterize acute membrane disintegration (lactate dehydrogenase (LDH) assay and live-cell PI uptake) and changes in mitochondrial membrane potential (ΔΨm) by the 5,5′,6,6′-Tetrachloro-1,1′,3,3′-tetraethylbenzimidazolyl-carbocyanine iodide (JC-1) assay. B[a]P did not cause an increase in extracellular LDH activity (Fig. 1f) or Propidium iodide (PI)-permeable plasma membrane leakiness (Fig. 1g) at either time point. At 24 h, reductions in ΔΨm were dose-dependent up to 0.25 µM B[a]P, but gradually returned to controls levels at treatments > 1 µM B[a]P. B[a]P treatment to doses ≥ 0.25 µM for 48 h caused significant reductions in ΔΨm to < 70% of control levels (Fig. 1h). Valinomycin, which served as a positive control of depolarization, reduced ΔΨm to 15.6% ± 2.9% of vehicle controls. Note that JC-1 results indicated mitochondrial dysfunction, whereas tetrazolium and resazurin methods suggested either enhanced proliferation or mitochondrial function.

To examine whether proliferation in Beas-2B was altered by B[a]P, a semi-quantitative analysis of cell cycle checkpoint regulators (p)-Chk1(S345), (p)-Chk2(T68), (p)-cdc2(Y15), and (p)-cdc2(T161) via immunodetection was performed. Compared to controls, 1 µM and 10 µM B[a]P induced significant elevations in (p)-Chk1(S345) (Fig. 1i) and was associated with an increase in the relative ratio of (p)-p53(S15)-to-p53 (Fig. 1j); no significant changes in (p)-Chk2(T68) and cdc-2 phosphorylation were observed (Fig. 1l). Beas-2Bs treated with 3 µg/mL aphidicolin (DNA synthesis inhibitor) resulted in significant elevation in (p)-Chk1(S345) immunodetection with no alteration in (p)-Chk2(T68) in agreement with previous studies^33–36^. Treatment with 100 ng/mL nocodazole (a beta-tubulin destabilizing agent) reduced (p)-cdc2(Y15), (p)-Chk1(S345), and (p)-Chk2(T68) (Fig. 1l), also in agreement with other published studies^37,38^. As a functional confirmation, the doubling time for Beas 2B cell growth was calculated from individual growth curves (Supplementary Fig. 5). The doubling time of untreated control as 22.2 h consistent with Costa, et al.^39^, while B[a]P dose-dependently increased doubling time, reaching 40 h with B[a]P treatments ≥ 5 µM (Fig. 1k).

**Figure 2:**
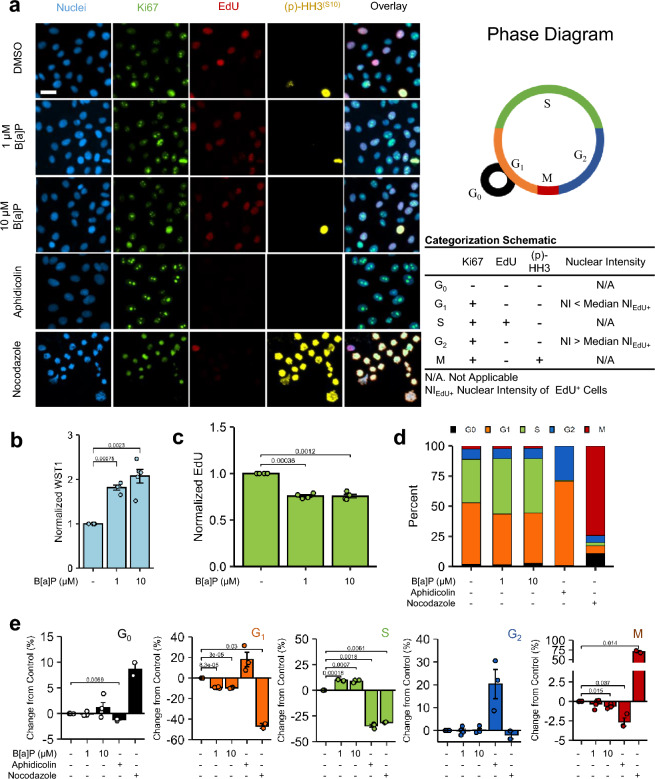
B[a]P-Induced Changes in Cell Cycle. (**a**) Beas-2B cells treated with 1 µM or 10 µM B[a]P (24 h), 3 µg/mL aphidicolin (18 h), and 100 ng/mL nocodazole (18 h) were prepared for cell imaging post-treatment and immunofluorescent staining. A reference cell cycle diagram is shown. The categorization schematic applied to immunofluorescent images is presented as well as a reference diagram of mammalian cell cycle. Scale bar = 25 μm. (**b**) Beas-2B cells treated with B[a]P were assessed for WST1 metabolism. n = 5. (**c**) Quantitative EdU uptake among EdU + Beas-2B cells treated with B[a]P. n = 4. (**d**) Cumulative cell cycle phase distributions of Beas-2B cells treated with B[a]P or cell cycle controls are presented. n = 4, except nocodazole-treated Beas-2B for which n = 2. (**e**) Magnitude changes compared to untreated controls among each categorical cell cycle phase are presented; bar colors coincide with phase designations presented in (**d**). n = 4, except nocodazole-treated Beas-2B for which n = 2. Positive changes denote increased percentage of cells in a specific phase among treated cells, whilst negative values denote decreased percentage. Endpoints are the arithmetic mean of listed independent experiments with error bars denoting one standard error of the mean.

Beas-2B cells treated with either 1 µM or 10 µM B[a]P for 24 h were assessed for differential cell cycle analysis via high-content imaging (Fig. 2a). A these B[a]P treatment concentrations, WST1 metabolism was significantly elevated as expected (Fig. 2b), while 5-ethynyl-2'-deoxyuridine (EdU) DNA incorporation, a marker of DNA replication, was significantly reduced by 23–25% compared to controls (Fig. 2c). Despite reductions in EdU uptake, BEAS-2B cell prevalence in S-phase, described as EdU + cells, was slightly elevated and G_1_ prevalence was significantly decreased compared to controls (Fig. 2e). No significant changes in prevalence were noted in G_0_ or G_2_ (Fig. 2e), while 10 µM B[a]P caused a slight, but significant, reduction in M phase. Aphidicolin and nocodazole arrested Beas-2B cells as expected. Phase prevalence breakdowns are shown in Supplementary Table S1 for reference.

### Benzo[a]pyrene and the pentose phosphate pathway

**Figure 3:**
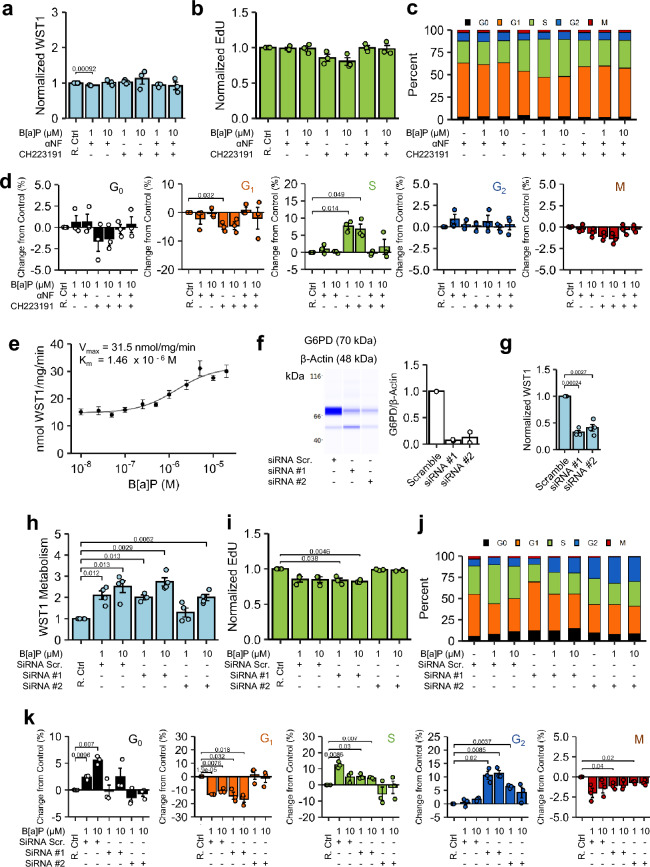
Effect of Inhibition of CYP1 Metabolic System. (**a–d**) Beas-2B cells treated with 1 µM or 10 µM B[a]P for 48 h in the presence of 10 µM aNF (CYP and AhR inhibitor), 5 µM CH223191 (AhR Inhibitor), or both simultaneously, followed by **(a)** WST1 metabolism, **(b)** EdU uptake in EdU + cells, **(c)** cumulative cell cycle phase quantitation, and **(d)** magnitude changes in each cell cycle relative to respective vehicle- and inhibitor-only control (R. Ctrl). n = 3. (**e**) Velocity of WST1 metabolism per unit protein per min was quantitated 48 h post-treatment; metabolism followed Michaelis–Menten kinetics. n = 3. (**f**) siRNA knockdown of G6PDH in Beas-2B cells was confirmed via semi-quantitative Western Analysis 96 h post-transfection. n = 2. Full-length capillary electrophoresis is presented in Supplementary Fig. 7 and the source of those images showed in Supplementary Fig. 11. (**g**) G6PDH-knockdown Beas-2B cells were assessed for WST1 metabolism 96 h post-transfection. n = 4. (**h–k**) Beas-2B cells treated with 1 µM or 10 µM B[a]P for 48 h after transfection with two different G6PDH siRNAs as well as a non-targeting scramble siRNA negative control. Following treatment, Beas-2B cells were assessed for (**h**) WST1 metabolism. n = 4. Beas-2B cells were then assessed for (**i)** EdU uptake in EdU + cells, (**j**) cumulative cell cycle phase quantitation, and** k**, magnitude changes in each cell cycle relative to respective vehicle- and inhibitor-only control (R. Ctrl). For clarity, normalized inhibitor controls are not presented individually, but represented by the first bar in each chart with “R. Ctrl”. Endpoints are the arithmetic mean of listed independent experiments with error bars denoting one standard error of the mean.

As stimulation of MTT, MTS, and WST1 metabolism were analogous, WST1 served as the indicator endpoint for inhibitor studies given its robust dynamic range of metabolic induction. Since B[a]P is a ligand of the AhR and a substrate of CYP450 isoforms, hyperstimulatory metabolic induction was potentially sensitive to B[a]P bioactivation and/or metabolic reprogramming. To address these, Beas-2B cells were pretreated with an AhR inhibitor,5 µM 1-Methyl-N-[2-methyl-4-[2-(2-methylphenyl) diazenyl]phenyl-1H-pyrazole-5-carboxamide (CH223191), a CYP1/AhR inhibitor,10 µM alpha-naphthoflavone (αNF), or both simultaneously, prior to treatment with 1 µM and 10 µM B[a]P for 48 h. Irrespective of B[a]P concentration, αNF and/or CH223191 abrogated B[a]P-induced WST1 hyperstimulatory metabolism and reductions in EdU uptake (Fig. 3a,b). Of note, B[a]P-induced WST1 hyperstimulatory metabolism was also reversed 24 h post-treatment (Supplementary Fig. 6). Despite efficacy in abrogating WST1 hyperstimulation, CH223191 alone failed to reverse significant B[a]P-induced shifts in cell cycle prevalence, while αNF completely reversed B[a]P-induced EdU and cell cycle changes to αNF-only treated controls (Fig. 3c,d).

Examination of B[a]P-induced hyperstimulatory metabolism of WST1 kinetically showed an monotonic metabolism curve that was explained sufficiently by Michaelis–Menten kinetics (Fig. 3e) and depended upon basal competency of the CYP1 metabolic machinery. Given the kinetic profile and WST1 dependency on reducing equivalents^21^, we reasoned G6PDH-derived NADPH was a likely contributor. Transfection of Beas-2B cells with 2 different G6PDH siRNAs resulted in > 80% reduction in basal protein (Fig. 3f) and > 50% reduction in basal WST1 metabolism (Fig. 3g) compared to negative non-coding siRNA scramble controls. Beas-2B treated with B[a]P in the presence of scramble siRNA showed profound hyperstimulatory WST1 metabolism that was largely unmitigated by G6PDH-targeting siRNA, except for Beas-2B cells treated with 1 µM B[a]P after siRNA #2 transfection. G6PDH siRNA #2 rescued Beas-2B cells from B[a]P-induced changes in EdU uptake and cell cycle changes (Fig. 3h–k); G6PDH siRNA #1 was remarkably similar to that of the negative siRNA scramble. Of note, both G6PDH siRNAs and the scramble tended to expand G_0_ prevalence compared to non-transfected cells, which was likely an artifact of transfection.

**Figure 4:**
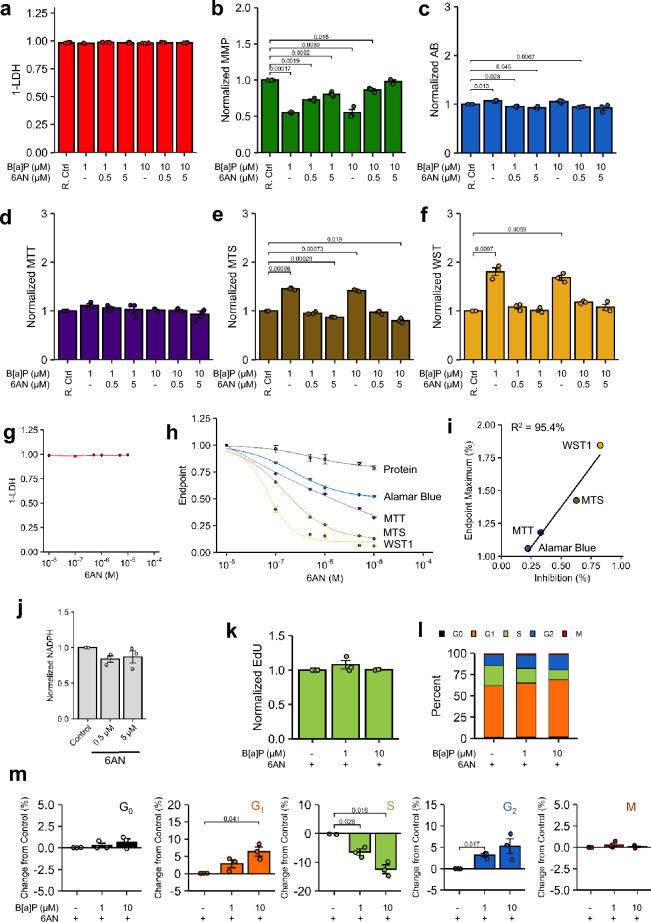
Endpoint-specific Sensitivity to 6-Aminonicotinamide. (**a-f**) Beas-2B cells treated with graded concentrations of 6AN and B[a]P for 48 h were assessed for (**a**) extracellular leakage via LDH and for (**b**) ΔΨm via JC-1, (**c**) Alamar Blue, (**d**) MTT, (**e**) MTS, and (**f**) WST1 metabolism. Vehicle- and inhibitor-only controls are represented by a unified group designated as R. Ctrl. n = 3. (**g**,**h**) Cells treated with graded concentrations of 6AN were assessed for cytotoxicity via (**f**) extracellular LDH leakage and (**g**) resazurin/tetrazolium endpoint metabolism as well as reduction in cell number as indicated by total protein recovery post-treatment; curve designations are annotated within the graph. n = 3. (**i**) Normalized endpoint metabolism induced by 10 µM B[a]P regressed against baseline endpoint-specific metabolic reduction by 0.5 µM 6AN demonstrated excellent linear correlation (Coefficient of correlation 95.4%). (**j**) Beas-2B cells treated with 6AN were assessed for NADPH content with correction for total protein. n = 3. (**k–m**) Beas-2B cells treated with 1 µM or 10 µM B[a]P for 48 h in the presence of 0.5 µM 6AN, followed by **(k)** EdU uptake in EdU + cells, **(l)** cumulative cell cycle phase quantitation, and **(m)** magnitude changes in each cell cycle relative to respective control. n = 3. Endpoints are the arithmetic mean of listed independent experiments with error bars denoting one standard error of the mean.

Genetic knockdown of G6PDH did not provide clear evidence of gene expression dependency, we assessed the same system using 6AN—a competitive inhibitor of G6PDH. Similar to 6AN alone, the co-treatment of 6AN and B[a]P did not induce plasma membrane permeability (Fig. 4a) or exacerbate ΔΨm reduction (Fig. 4b). Paradoxically, 5 µM 6AN in combination with 10 µM B[a]P returned ΔΨm to control levels. For all tetrazolium salt and Alamar Blue assays, 6AN categorically blocked B[a]P-induced hyperstimulation 48 h-post treatment (Fig. 4c–f) as well as at 24 h post-treatment (Supplementary Fig. 8). Despite no lytic cytotoxicity across the broad range of 6AN tested (Fig. 4g), 6AN dose-dependently inhibited metabolism of all endpoints tested compared to vehicle-treated controls and replicated the hierarchy of B[a]P-induced hyperstimulation that was previously observed: WST1 > MTS > MTT > Alamar Blue (Fig. 4h). 6AN IC_50_ values for each metabolic endpoint are presented in Table 1. Normalized against total protein, regression of 10 µM B[a]P-induced endpoint-specific hyperstimulatory metabolism against assay-specific total protein-normalized metabolic inhibition by 0.5 µM 6AN yielded a linear correlation (R^2^ = 95.4%; Fig. 4i), though 6AN did not significantly lower intracellular NAPDH compared to controls (Fig. 4j). Analogously to αNF, 0.5 µM 6AN abrogated B[a]P-induced changes in EdU uptake (Fig. 3i) and cell cycle changes (Fig. 4k,m).

**Table 1:** Modeled 6-Aminonicotinamide IC_50_ Values.

Endpoint	IC50 (M)	CI95% (M)
Protein	> 10 × 10^–5^*	> 10 × 10^–5^*
Alamar Blue	> 10 × 10^–5^*	> 10 × 10^–5^*
MTT	4.1 × 10^–6^	2.7 × 10^–6^–5.4 × 10^–6^
MTS	1.6 × 10^–7^	1.5 × 10^–7^–1.7 × 10^–7^
WST1	6.9 × 10^–8^	5.7 × 10^–8^–8.1 × 10^–8^

*6-Aminonicotinamide did not reduce endpoint below 50% relative to vehicle controls up to the highest concentration of > 10^–5^ M.

**Figure 5:**
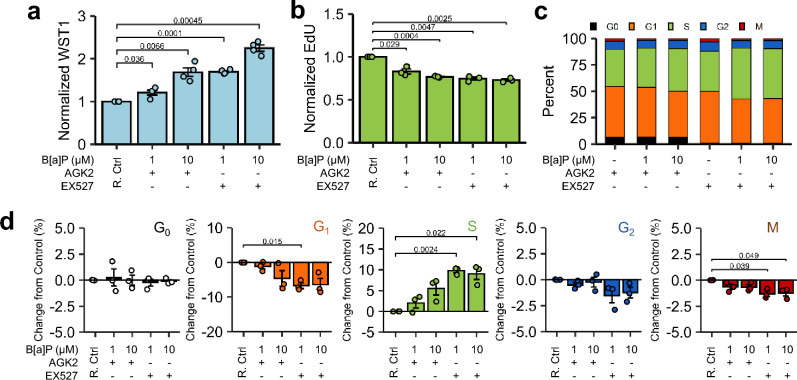
Endpoint-specific Sensitivity to SIRT Inhibitors. (g–j) Beas-2B cells treated with 1 µM or 10 µM B[a]P for 48 h in the presence of 10 µM AGK2 (SIRT2 Inhibitor) or 0.5 µM EX527 (SIRT1 Inhibitor), followed by (**a**) WST1 metabolism, (**b**) EdU uptake in EdU + cells, (**c**) cumulative cell cycle phase quantitation, and (**d**) magnitude changes in each cell cycle relative to respective vehicle- and inhibitor-only control (R. Ctrl). n = 3. Endpoints are the arithmetic mean of listed independent experiments with error bars denoting one standard error of the mean.

Since CYP450 metabolism depends, at least in part, on PPP-derived NADPH, we examined whether G6PDH metabolic competence was the source of B[a]P-induced hyperstimulatory metabolism across all resazurin and tetrazolium endpoints. Since G6PDH phosphorylation by Sirtuin-2 (SIRT2) is known to enhance G6PDH catalytic activity^40,41^, we examined whether SIRT2 contributed to the effects observed. Unlike 6AN, AGK2 (SIRT2 inhibitor) reversed B[a]P-induced effects at 1 μM, but not 10 μM concentration, while the Sirtuin-1 (SIRT1) inhibitor, EX527, expectedly did not reverse observable WST1 metabolism or cell cycle changes (Fig. 5a–d).

## Discussion

**Figure 6:**
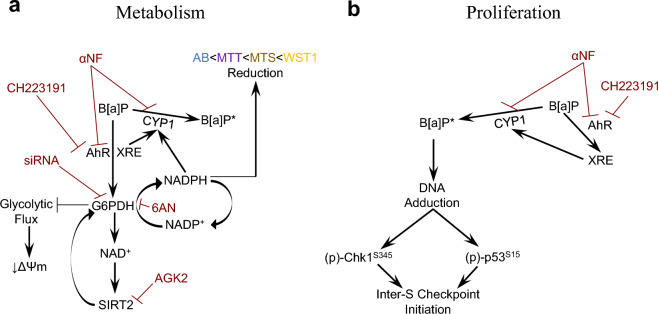
Proposed mechanism for B[a]P-induced hyperstimulatory metabolism and proliferative changes in Beas-2B cells. (**a**) B[a]P induces a metabolic shift from glycolysis to the Pentose Phosphate Pathway mediated by XRE-related transcription. Nucleotide ratio imbalance triggers post-translational modification of G6PDH by SIRT2 and transcriptional regulation by the XRE combine to restore NADPH levels required to supply microsomal metabolism of B[a]P, leading to reduction in glycolytic flux and transient reductions in mitochondrial membrane potential. (**b**) Constitutive CYP1 bioactivates B[a]P to a genotoxic metabolite, while B[a]P perpetuates CYP1 expression through XRE. Enhanced CYP1 metabolism further contributed to genotoxic insult, resulting in transient inter-S checkpoint initiation mediated through Chk1 and p53. Abbreviations: AhR, Aryl Hydrocarbon Receptor; B[a]P, Benzo[a]pyrene; CYP1, Cytochrome P450 Isoforms 1A1 and 1B1; G6PDH, Glucose-6-Phosphate Dehydrogenase. NAD+, Oxidized Nicotinamide Adenine Dinucleotide; NADP+, Oxidized Nicotinamide Adenine Dinucleotide Phosphate; NADPH, Reduced Nicotinamide Adenine Dinucleotide Phosphate; (p)-Chk1^S345^, Phospho-Checkpoint Kinase 1; (p)-P53^S15^, Phospho-P53; SIRT2, Sirtuin 2; XRE, Xenobiotic Response Element; ΔΨm, Mitochondrial Membrane Potential. 6AN, 6-Aminonicotinamide; AGK2, 2-cyano-3-[5-(2,5-dichlorophenyl)-2-furyl]-N-5-quinolinylacrylamide; CH223191, 1-Methyl-N-[2-methyl-4-[2-(2-methylphenyl)diazenyl]phen yl-1H-pyrazole-5-carboxamide; siRNA, Silencing RNA; αNF, Alpha-Naphthoflavone. AB, Alamar Blue; MTT, 3-(4,5-Dimethylthiazol-2-yl)-2,5-diphenyltetrazolium bromide; MTS, 3-(4,5-dimethylthiazol-2-yl)-5-(3-carboxymethoxyphenyl)-2-(4-sulfophenyl)-2H-tetrazolium; WST1, 3-(4,5-dimethylthiazol-2-yl)-5-(3-carboxymethoxyphenyl)-2-(4-sulfophenyl)-2H-tetrazolium.

The current investigation aimed to offer insight into the underlying sources of metabolism of routine tetrazolium and resazurin agents used in cytotoxicity and proliferation characterization. We used B[a]P as an inducer of the PPP^42^ and 6AN as an inhibitor of G6PDH^43,44^ to interrogate the contributory metabolism of the PPP on routine cytotoxicity methods in Beas-2B cells. Present results demonstrated that the enhanced cellular metabolism of routine tetrazolium salts and Alamar Blue upon treatment with B[a]P did not irrevocably implicate either “mitochondrial activity” or “proliferation”—interpretations that remain pervasive in the in vitro toxicology literature^45–47^—but rather an aggregate of biological processes, including PPP-derived NADPH (Fig. 6). While this observation has been noted previously for the PPP^21^, no investigation has examined differential sensitivity of methods presented to a specific biological process in such detail, as well as attempt to examine the underlying modulators of G6PDH-associated metabolism.

The general practice with toxicological in vitro assessment necessitates the confirmatory testing of cytotoxicity/proliferation endpoints using at least one secondary method of a differing mechanistic class, i.e., confirmation of cytotoxicity measured via tetrazolium salt method using a membrane permeability assay. For a given laboratory, LDH typically serves as the confirmatory test for cytotoxicity, while several methods for assessing DNA synthesis, e.g., EdU or BrdU, etc., may be employed to verify proliferation. However, two issues arise during confirmatory testing: (1) appropriateness of specific cytotoxicity assessment employed in the model system and (2) ultimate interpretation of biological effect when inter-assay discordance arises. To the former point, inadequate assessment of assay-specific technical interference for one or both employed method(s) undermines the validity of cytotoxicity/proliferative interpretation^48^, especially with nanomaterials that are particularly sensitive to interference^49^. While this interference is pervasive for nanomaterials^50^, small molecule interference is also common^16,51^. In pursuant of discordant results, reliance upon a limited selection of confirmatory cytotoxicity assays remains pervasive and relies generally on measurement of LDH release or ATP-derived endpoints^52–56^. Compounding this issue, significant gaps in knowledge remain about mechanisms of resazurin and tetrazolium metabolism, even though several groups have contributed significantly to the understanding of these endpoints^57–60^. Initially, our laboratory had observed robust hyperstimulatory WST1 metabolism during cytotoxicity screening of incinerated thermoplastic samples^61^ similar to those observed with organic extracts of diesel exhaust particles^62^. Zhu et al.^63^ observed that micromolar B[a]P concentrations induced hyperstimulatory MTT metabolism similar to the current investigation, thus prompting this examination.

Initial observations of WST1 hyperstimulatory tetrazolium metabolism with B[a]P treatment, in the absence of extracellular LDH leakage or PI uptake, suggest enhanced proliferation. Using another tetrazolium salt or resazurin failed to recapitulate the magnitude of metabolic induction measured with WST1 (Fig. 1). Conflicts between cytotoxicity measures for metabolic endpoints, such as tetrazolium salts, and cell-by-cell methods, such as nuclear propidium iodide staining, are known for cytotoxic agents^64^, thus multiple additional measures of proliferation and cytotoxicity were performed. Immunodetection analysis, doubling time, and cell cycle immunofluorescence (Fig. 2) negated the interpretation of the observed hyperstimulatory metabolism as purely proliferation, as suggested by the significant reduction in the rate of passage through S-phase^65^. Coupled with elevated (p)-Chk1(S345) and a (p)-p53-to-total p53 ratio via immunodetection analysis and an absence of stark changes in G_2_ or M-phase prevalence (Figs. 1 and 2), these results indicated transient induction of the inter-S checkpoint due to B[a]P, likely through ATM/ATR axis^66^. The results confirmed observations from similarly designed studies in which B[a]P increased the prevalence of S-phase in this cell line^41,63^. With insignificant changes in G_2_ prevalence or cdc2 phosphorylation at the tyrosine-15 residue, the results are inconsistent with induction of the G_2_/M checkpoint^67,68^. CH223191, a competitive inhibitor of AhR, alone failed to block reductions in EdU uptake, which could be explained as either (1) basal metabolic machinery in Beas-2B cells produced enough genotoxic metabolite of B[a]P^69^ to initiate inter-S checkpoint or (2) B[a]P and/or its metabolites can activate other nuclear receptors involved in CYP induction and perpetuation of genotoxic stress^70^. While 6AN inhibited B[a]P-induced effects on EdU uptake, co-treatment led to a paradoxical reduction in S-phase prevalence compared to 6AN treatment-only controls and was larger in magnitude than the effects observed by G6PDH siRNA #2. Together, these results indicate that the activation of the inter-S checkpoint in Beas-2B cells impinged upon an intact CYP450 machinery which included not only CYP metabolism but also AhR and NADPH. Further research is required to attribute the relative contribution of each portion of the system.

AGK2 partially attenuated B[a]P effects on EdU uptake, while inhibition of CYP1/AhR by αNF, G6PDH catalytic activity by 6AN, and G6PDH siRNA #2, categorically reversed B[a]P-induced cell cycle changes. EX527 failed to alter the response, as expected. Since AGK2 increases G6PDH catalytic activity post-translationally via SIRT2^40,41^, blocking SIRT2 did indeed partially reverse hyperstimulation indicating SIRT2 and, thus, likely plays a role in the B[a]P bioactivation response (Fig. 5). Since EX527 is not associated with post-translational modification of G6PDH, its inability to modulate the B[a]P response was expected. Other known cytosolic sources of NADPH, such as isocitrate dehydrogenase-1 (IDH1), malic enzyme-1 (ME1), and other compartmental stores^22–24,71^, could not generate NADPH in sufficient concentrations to sustain microsomal metabolism for B[a]P. Ultimately, the basal microsomal metabolic machinery and PPP-derived cytosolic NADPH concentrations in Beas-2B cells were sufficient to initiate and maintain genotoxic stress by B[a]P metabolites. G6PDH catalytic enhancement by SIRT2 likely initiated a feed-forward loop but does not seem to account for the entire regulatory mechanism of G6PDH; the exact contribution of SIRT2 under basal conditions are less clear but warrants further investigation. Furthermore, a myriad of other regulators of G6PDH are known, including ATM^72^, whose contributions in this system were not explored, and may explain only partial attenuation by AGK2. Examination of proliferative capacity discounted an interpretation of proliferation as the source of hyperstimulatory metabolism observed in MTS and WST1 assays.

To establish a link between resazurin/tetrazolium metabolism and their respective dependence upon the PPP, we examined the contributory effects of proliferation, cytotoxicity, and assay interference to isolate the effect of the PPP on each of the endpoints tested. Here, we demonstrated that B[a]P did not interfere with the assay components, analyte chromophore, or detection of wavelength transmittance (optical activity) of the endpoints examined, thus they were non-contributory to the observed elevation in optical density (Supplementary Fig. 9). The nano- to low-micromolar concentrations of B[a]P did not induce significant lytic cytotoxicity in concordance with previous studies using Beas-2B cells^63,73–75^. Examination of high-content imaging (Fig. 2; Supplementary Fig. 10 and Supplementary Fig. 11) used for cell cycle analysis did not suggest substantial apoptosis induction at frequencies enough to significantly alter the interpretations drawn, thus underscoring the major effect of B[a]P on alterations in proliferation rate. 6AN dose-dependently reduced metabolism of the measured endpoints listed in order of magnitude: WST1 > MTS > MTT > Alamar Blue without overt cytotoxicity. The hierarchy of metabolic inhibition was linearly correlated with B[a]P-induced metabolic induction of each endpoint, thus providing correlative evidence of endpoint-specific sensitivity to the metabolic status of G6PDH. This correlation was supported by co-incubation with 6AN (Fig. 4h), further supporting each endpoint’s sensitivity to PPP-derived NADPH. These results are not perplexing as several studies implicate NADPH in the metabolism of WST1^76^, MTS^4^, and MTT^6^, though strict comparisons between each method have not been made. The contribution of water-soluble tetrazolium reliance may be due to differential reduction potentials among the two-electron cycling substrates used to enhance tetrazolium development (5-methylphenazium methyl sulfate for MTS and 1-methoxy-5-methylphenazium methyl sulfate for WST1). Further testing would be required to confirm differential reduction potentials among these cycling substrates. Nevertheless, at the most extreme, WST1 was almost exclusively metabolized by PPP-derived NADPH, followed by MTS, and then MTT; Alamar Blue was comparatively insensitive to NAPDH-derived sources in this model. Overall, CYP450 translational control and G6PDH competitive inhibition proved universally effective in abrogating B[a]P-mediated metabolic induction. These results demonstrated that, while the PPP was the source of reducing equivalents responsible for metabolic induction, an intact metabolic machinery, i.e., CYP450, of a defined enzymatic capacity was also required to precipitate a state of enriched PPP-derived NAPDH that could be readily reduced by resazurin and tetrazolium protochromophores.

We observed significant ΔΨm depolarization in B[a]P-treated cells compared to controls (Fig. 1). Generally, JC-1-reported ΔΨm depolarization is positively correlated with markers of cytotoxicity *in vitro*^77–80^; however, this was not presumed in the current investigation. The observed peak depolarization occurred between 1 and 10 µM B[a]P (48 h exposure)—the dose range associated with maximal hyperstimulatory metabolism of all tested tetrazolium salt methods. If tetrazolium reduction was correlated with mitochondrial function, as remains contemporaneously purported^45–47^, then one would expect significant reductions in tetrazolium metabolism in association with ΔΨm depolarization. To this point, the authors purport the changes in ΔΨm, including 6AN-mediated apparent attenuation of ΔΨm depolarization by B[a]P, directly support an active B[a]P-induced metabolic shift from glycolysis to PPP as has been previously observed in RT4 bladder cancer cells using an untargeted metabolomic approach^42^. Results of the current investigation suggest redirection of glucose equivalents from glycolysis to the PPP to support NADPH-dependent microsomal systems by B[a]P. Additionally, fructose-6-phosphate (F-6-P), an intermediate of the PPP, typically undergoes further glycolytic metabolism to eventually enter the TCA cycle to support retention of ΔΨm. However, F-6-P can be metabolized back to G-6-P for re-entry into the PPP. Given the ΔG° for conversion of G-6-P to F-6-P is slightly positive^81^, the reverse reaction can become thermodynamically favorable as to become spontaneous, especially if G-6-P concentrations are kept sufficiently low through rapid tonic metabolism by G6PDH. This is the most plausible explanation for the metabolic data in the current investigation, especially since B[a]P-induced WST1 metabolism can be explained sufficiently by Michaelis–Menten kinetics. This suggests a single rate-limiting step that constrained the metabolic induction rate in the present system. Further studies would be required to confirm these assertions as well as to identify this presumed rate-limiting step in the observed process. The present results in this model offer more evidence to support previous assertions that mitochondrial function and tetrazolium metabolism can be uncoupled in viable cells^6,15,82^.

Based on the presented Beas-2B cell proliferation determinations and resazurin/tetrazolium salt metabolic data, B[a]P induces a metabolic shift from glycolysis to the PPP. This shift was detected most sensitively via water-soluble tetrazolium methods (WST1 and MTS), while MTT and Alamar Blue showed minimal metabolism induction in this system. Considering only the observed hyperstimulatory WST1 metabolism and lack of B[a]P-induced LDH release without context could erroneously lead to a conclusion of enhanced cell proliferation. However, the present results demonstrate that the magnitude of B[a]P-induced assay metabolism is strictly associated with sensitivity to PPP inhibition, thus warranting preliminary validation in method utilization for agents which may perturb PPP metabolism. Without complete examination of the nuanced method-specific heterogeneities among routine tetrazolium/ resazurin methods utilized in toxicity testing, qualifying results of specified cytotoxicity measures under test conditions become untenable. For example, a choice confirmatory test of LDH would have failed in recognizing the reduced proliferative capacity of B[a]P-treated Beas-2B cells. Therefore, delineating and weighing discrepancies between screening and confirmatory testing becomes equally untenable, leading to uncertainties in rationalizing cytotoxic outcomes. As shown, investigations examining cytotoxic and proliferative changes due to AhR ligands, such as PAHs, should consider the potentially confounding contribution of metabolic reprogramming. Further, methods for even routine cytotoxicity detection which rely on NADPH-mediated reduction should be preliminarily validated for potential interactions from PPP metabolism prior to applications under test conditions.

## Methods

### Cell culture and treatment exposures

Beas-2B cells, human bronchial epithelial cells which have been widely used for in vitro cytotoxicological studies, were purchased from ATCC (CRL-9609; Manassas, VA) and maintained in complete airway epithelial growth medium (AEGM; Promocell, GmbH; Heidelberg, Deutschland) in a humidified environment at 37 °C with 5% v/v CO_2_ in air. Cells were cultured in vented tissue culture flasks and passaged according to standard practice. All experiments were performed on cells between the 8th and 14th passages from vendor receipt (Passage 38) to control for phenotypic drift. Beas-2B were plated at 5,000 cells/well in 96-well plates, 20,000 cells/well in 24-well plates, or 50,000 cells/well in 6-well plates 48 h prior to exposure with B[a]P (Cat. 51,968; Millipore-Sigma; St. Louis, MO). Dose–response concentrations ranged from 2.5 nM to 20 μM (2.5 × 10^–8^ to 2 × 10^–5^ M); DMSO-treated controls were designated as 10 nM (1 × 10^–8^ M) on semi-logarithmic charts. Further studies were conducted using either 1 or 10 μM. Groups plated for doubling time analysis were plated at a density of 25,000 cells/well in 6-well plates. Cells intended for immunofluorescent imaging, mitochondrial membrane potential (ΔΨm), and Alamar Blue analyses were plated in tissue culture-treated 96-well black-wall microplates; cells designated for colorimetric assays were plated in clear microplates. All tissue culture-treated plasticware were purchased from Corning Inc. (Corning, NY).

Molecular inhibitors used in this investigation were: CH223191 (Cat. C8124; Millipore-Sigma), alpha-naphthoflavone (αNF; Cat. N5757; Millipore-Sigma), 6-aminonicotinamide (6AN; Cat. A68203; Millipore-Sigma), AGK2 (Cat. 13,145; Caymen Chemical; Ann Arbor, MI), and EX527 (Cat. A4181; APExBIO; Houston, TX). Detailed specifics on the inhibitors used are included in Supplementary Table S2. Inhibitors were pre-incubated one hour prior to B[a]P exposure and maintained throughout the duration of B[a]P exposure. As B[a]P and all inhibitors used are highly soluble in DMSO (Cat. D2650; Millipore-Sigma), the final concentration of DMSO was kept below 0.1% v/v in culture medium among all experiments, and 0.1% v/v DMSO only is used as treatment and inhibitor vehicle controls.

### Tetrazolium salt and alamar blue assays

Beas-2B cells were assessed 24- and 48-h post-exposure with WST1 (Cat. 11644807001; Millipore-Sigma), MTS (Cat. G3582; Promega Corporation; Madison, WI), MTT (Cat. M2128; Millipore-Sigma), and Alamar Blue (Cat. Y00100; Thermo Fisher Scientific, Inc.; Waltham, MA). At the end of each exposure, growth medium was changed once with fresh, pre-warmed medium prior to reagent addition and incubation under a humidified atmosphere at 37 °C with 5% v/v CO_2_ in air. All resazurin and tetrazolium salt endpoint dose–response curves, with exception of MTT-reported B[a]P dose–response (Fig. 1c), were modeled using the “drc” package in R Program version 3.6.3 (R Project for Statistical Programming; Vienna, Austria). Method-specific parameters are given in Table 2.

**Table 2:** Tetrazolium and alamar blue assay conditions.

Method	Proportion dye:medium	Incubation duration	Quantitation method
WST1	1:9	2 h	Absorbance^1^ Measure: 450 nm Reference: 650 nm
MTS	1:4	2 h	Absorbance^1^ Measure: 490 nm
MTT	1:4	4 h *	Absorbance^1^ Measure: 570 nm Reference: 600 nm
Alamar Blue	1:9	3 h	Fluorescence^2^ Excitation: 560 Emission: 590

*Insoluble MTT formazan was resolubilized in neat DMSO

^1^SpectraMax Plus 384 and SoftPro Max data acquisition software (Molecular Devices; Le Jolla, California)

^2^Varioskan LUX and SkanIt v. 4.1 data acquisition software (Thermo Fisher Scientific, Inc.).

WST1 metabolic velocity was calculated via Beer’s Law assuming a molar extinction coefficient of 37,000 M^−1^*cm^−1^ as described by^2^. Briefly, 2 mL of WST1 formazan was produced using untreated cells under standard conditions described above. Aliquots of WST1 formazan-containing medium and respective blank optical density were measured simultaneously in a 1 cm polystyrene cuvette and in a clear bottom microplate (A450-A650) containing 110 µL (100 µL AEGM + 10 µL WST1) to correlate optical density values in microplate format to cuvette-derived WST1 formazan concentration values in nmol WST1 formazan/L/2 h. Calculated well-specific formazan concentrations from microplate assays were divided by 60 min/hour and accompanying well-specific whole-cell lysate protein concentration as derived by BCA in mg protein/L to convert the kinetic metabolic rate to units of nmol WST1 per minute per mg total protein (nmol WST1/mg/min); this method assumes WST1-to-formazan conversion is both irreversible and does not reach complete WST1 conversion in 120 min. AEGM-only containing wells were processed analogously to Beas-2B containing wells and served as the blank to account for protein left behind by the culture medium.

### Interference testing

Interference testing was conducted in two acellular conditions. Interaction of B[a]P with tetrazolium and Alamar Blue during reagent development (Assay Interference) was examined by incubating working reagents under typical incubation conditions (Table 2) in the presence of B[a]P-containing medium. Interaction of B[a]P with the metabolic end-product of each respective reagent (Product Interference) was evaluated, with exception of MTT. Fresh end-product was attained by batch metabolizing each endpoint assay in T25 flasks containing Beas-2B cells. Medium was then abstracted and centrifuged to remove cells prior to B[a]P addition to supernatants at final concentrations analogous to cell treatments. End-product-B[a]P mixtures were plated in respective 96-well microplates and incubated under culture conditions for each method’s respective incubation duration noted in Table 2 prior to quantitation. Since B[a]P-containing medium was aspirated prior to DMSO resolubilization, product interference was not performed for the MTT assay. Final concentration of B[a]P was assumed to contribute negligibly to optical activity as demonstrated Assay Interference testing. B[a]P caused negligible technical interference with assay reagents and formazan products (Supplementary Fig. 9). Since B[a]P-containing media was changed prior to assessment during testing, technical interference by B[a]P was considered non-contributory to observed changes in endpoint metabolism.

### Mitochondrial membrane potential

Mitochondrial membrane potential (ΔΨm) was measured via JC-1 ratiometric analysis. Immediately following indicated post- exposure, AEGM was replaced with fresh AEGM, and the cells were stained for 20 min with 1 µg/mL JC-1 (Thermo Fisher Scientific, Inc.) under a humidified atmosphere 37 °C with 5% v/v CO_2_ and air. Beas-2B cells were gently washed twice with pre-warmed AEGM and held in fresh AEGM. Treatment with 10 µM valinomycin (Thermo Fisher Scientific, Inc.) for 30 min post JC-1 staining served as a mitochondrial membrane dissipation control. JC-1 Red:Green ratiometric analysis was quantitated using the SpectraMax M4 ELISA plate reader with SoftMax Pro v. 6.2.1 data acquisition software using excitation/emission wavelength pairs of 535/590 nm and 485/530 nm to measure J-aggregates (Red) and J-monomers (Green), respectively.

### Membrane permeabilization: lactate dehydrogenase and live-cell imaging

Two hours prior to Lactate Dehydrogenase (LDH) assessment, a set of wells containing untreated Beas-2B cells was treated with 1% v/v Triton X-100 as the 100% LDH release endpoint. Simultaneously, another group of Beas-2B cells was treated with 0.005% v/v Triton X-100 for necrotic gating in live-cell imaging. Following exposure, microplates were centrifuged for 5 min at 800 × rpm to pellet floating Beas-2B cells, and 50 µL of treatment medium abstracted to a new microplate pre-filled with 50 µL fresh medium per well. LDH was detected as per manufacturer’s instructions (Roche), with quantitation via absorption spectrophotometry at 490 nm and a reference wavelength of 650 nm using the SpectraMax Plus 384 and SoftMax Pro v. 5.4.1 data acquisition software (Molecular Devices). Untreated controls were designated as spontaneous LDH release for ascertainment of background. Values are expressed as percent LDH with lower and upper bounds designated as spontaneous and 100% LDH release, respectively.

Medium of treated Beas-2B sampled for LDH measurement was replaced with fresh pre-warmed medium and stained with 1 µM Hoechst 33,342 (Thermo Fisher Scientific, Inc.) and 5 µg/mL propidium iodide (PI; Thermo Fisher Scientific, Inc.) for 30 min under culture conditions prior to live-cell imaging. Microplates on the ImageXpress Micro XLS high-content imaging platform were imaged with MetaXpress V.6 image acquisition and analysis software (Molecular Devices). Specifications for the ImageXpress Micro XLS imaging platform are included in Supplementary Tables S3–S5. Treatment groups were imaged at 4 sites per well at 10$\times$ magnification across technical replicate wells and aggregated. Images were then analyzed by multi-wavelength scoring for nuclei imaged using a DAPI filter set and necrotic cells via detection of PI using a Cy5 filter set. Nuclei were identified via intensity threshold- and size-based autodetection, and a binary mask applied to identify nuclear boundaries. Necrotic and viable cells were dichotomously categorized as necrotic (PI+) or live (PI−) through intensity-based gating. Beas-2B treated with 0.005% Triton X-100 served as a gating control for positive necrosis. Approximately 2–4% of untreated controls were routinely designated as PI+.

### Doubling time

To examine short-term proliferative capacity, Beas-2B cells were plated in 6-well microplates and treated with graded concentrations of B[a]P for 72 h. Cells were washed with fresh medium, passaged analogously to culture maintenance, counted using the Countess Automated Cytometer (Invitrogen) with 0.4% Trypan Blue, and re-plated in 96-well microplates at 1,000 viable cells/well. Cells were assessed for WST1 metabolism at 2 h and every 24 h thereafter up to 120 h. Doubling time (µmax^−1^) was derived from blanked WST1 curves using the package “growthrates” in the R Program version 3.6.3.

### Cell cycle

Unsynchronized Beas-2B cells were plated and treated as described above. One hour prior to treatment termination, cells were pulsed for 1 h with 5 µM 5-ethynyl-2'-deoxyuridine (EdU; Thermo Fisher Scientific, Inc.) prior to fixation with 4% paraformaldehyde in Dulbecco’s modified phosphate-buffered saline (DPBS, 20 min), permeabilization with 0.5% v/v Triton X-100 in dH2O (15 min), and holding in DPBS until subsequent multi-plate batch staining. EdU was detected via a Click-IT® labeling using an Alexa 647-conjugated azide as per manufacturer’s instructions (Cat. C10419; Thermo Fisher Scientific, Inc.). Cells were washed thrice with DPBS and blocked in 1% w/v BSA in DPBS for 1 h at room temperature. Nuclear targets were probed using a mouse anti-Ki-67 (Cat. 9449; Cell Signaling Technology; Danvers, MA; Dilution 1:400) and rabbit anti-phosphorylated-Histone H3[S10] ((p)-HH3; Cat. 53,348; Cell Signaling Technology; Dilution 1:400) antibodies in blocking buffer for 1 h. Nuclear targets were then simultaneously immunodetected with anti-rabbit-Alexa-555 (Cat. 4413; Cell Signaling Technology; Dilution 1:600) and anti-mouse-Alexa-488 (Cat. 4408; Cell Signaling Technology; Dilution 1:600) secondary F(ab’)_2_ antibodies and nuclei counterstained with 1 µM Hoechst 33,342 for 1 h in blocking buffer. Stain-free background controls were included that underwent the complete staining procedure with exception of EdU pulse labeling and primary antibody immunodetection. Microplates held in DPBS were imaged 4 sites per well at 20X magnification across technical replicate wells using the ImageXpress Micro XLS platform. Analogously to live-cell imaging, nuclei were identified and masked for establishment of nuclei boundaries for DNA content quantitation as well as identification of nuclear EdU (S Phase), Ki-67 (G_1_-S-G_2_-M Phases), and (p)-HH3 (M Phase) using the Custom Module Editor in the MetaXpress v.6 Software. Determination of threshold autofluorescence using unstained controls allowed for positivity masking for EdU, Ki-67, and (p)-HH3 nuclear markers, followed by mask centroid reduction, centroid/nuclear mask filtering, and positivity scoring to reduce inter-nuclear crossover resulting in false-positives. An additional binary mask was applied to EdU-positive nuclei, allowing for intensity-derived EdU uptake. Cell cycle designations were attributed to each cell detected using a combination of Hoechst 33,342-enabled cell cycle assessment refined by phase-specific markers. The phase determination schematic is presented in Fig. 2A. Cell cycle data presented were calculated as changes from control.

### Western blot immunodetection

Beas-2B cells treated with B[a]P for 24 h were washed twice with 1X DPBS and lysed in radio immunoprecipitation (RIPA) buffer (Millipore-Sigma) containing 1X protease inhibitors (Millipore-Sigma), 1 mM phenylmethylsulfonyl fluoride (Millipore-Sigma), and 1 mM sodium orthovanadate (Millipore-Sigma) on ice for 5 min with gentle agitation. Whole-cell lysates were collected in microcentrifuge tubes and ultrasonicated using an ultra-fine tip sonicator (Vibra-Cell) for 5 s. Insoluble debris were pelleted by centrifuging sonicated lysates at 13,000 × g for 10 min at 4 °C, and supernatant removed to a fresh tube. An aliquot of whole-cell lysate was abstracted and diluted 1:10 in DPBS for protein quantitation via the BCA method (Cat. 23225; Thermo Fisher Scientific, Inc.).

Western Blot analysis was performed using the Wes Automated Immunoblot Platform (ProteinSimple; San Jose, CA). Antibodies used for Western Blotting analysis were purchased from Cell Signaling Technology, except where noted. All reagents utilized in Western Blot analysis were acquired from ProteinSimple. Briefly, whole-cell lysates were diluted to 1 mg/mL in 0.1X assay buffer, and combined with a 5X fluorescent Master Mix prior to boiling for 5 min at 95 °C. Lysates were allowed to cool to room temperature, centrifuged for 5 min at 1,000 × g at room temperature, and loaded into a reagent plate (ProteinSimple). Phospho-cdc2^Tyr15^ ((p)-cdc2(Y15); Cat. 4539), phospho-cdc2^Thr161^ ((p)-cdc2(T161); Cat. 9114), phospho-Chk1^Ser345^ ((p)-Chk1(S345); Cat. 2348), phospho-Chk2^Thr68^ ((p)-Chk2(T68); Cat. 2661), p53 (Cat. 2527), and β-actin (Cat. 3700) were diluted 1:50. Phospho-p53^Ser15^ ((p)-p53(S15); Cat. AF1043) was purchased from R&D Systems (Minneapolis, MN) and diluted 1:33. Glucose-6-phosphate dehydrogenase (G6PDH; Cat. NBP2-22125) was purchased from Novus Biologicals (Littleton, CO) and diluted 1:50. Primary antibodies were detected using either an HRP-conjugated anti-rabbit secondary antibody (Cat. 7074; Cell Signaling Technology) or an HRP-conjugated anti-mouse secondary antibody (Cat. 7076; Cell Signaling Technology) diluted 1:100. Band staining intensity was quantitated using the Wes by chemiluminescent detection and normalized against total protein as the loading control, except for confirmation of G6PDH siRNA knock-down that was normalized to β-actin. Data reduction was performed using the Compass Software (Compass for SW 4.0; ProteinSimple), yielding peak area derived from Gaussian-fitted curves to identified peaks.

### Intracellular NADPH and protein lysate quantitation

Beas-2B cells were plated at 2,500 cells/well in 96-well microplates. Cells were pretreated with 0.5 µM or 5.0 µM of 6AN prior to treatment with either DMSO or 0.5 µM B[a]P. NADPH was measured with the NADP/NADPH-Glo™ Assay according to manufacturer’s instructions (Cat. G9081; Promega Corporation). After derivatization of NADPH samples, 50 µL were abstracted and developed in an equal volume of freshly prepared NADP/NADPH-Glo™ detection reagent at 25 °C for 30 min. Samples were quantitated for luminescence using the Varioskan LUX with a 250-ms integration time using the SkanIt v. 4.1 data acquisition software. A set of Beas-2B cells treated simultaneously with NADP+/NADPH samples were lysed in RIPA Buffer without protease inhibitors. Lysates were quantitated using the BCA method to standardize sample luminescence detection with total lysate protein.

### G6PDH siRNA transfection

Beas-2B cells reverse transfected with siRNA at an initial seeding density of approximately 60–80% confluent. All liquid transfer devices, containers, and materials utilized for siRNA transfection were treated with RNAaseZAP (Thermo Fisher Scientific, Inc.); pipettes were equipped with aerosol-barrier tips to deter contaminating RNAses. Stock solution of Lipofectamine RNAiMAX concentration were made by diluting 12 µL Lipofectamine RNAiMAX (Thermo Fisher Scientific, Inc.) into 200 µL Opti-MEM (Thermo Fisher Scientific, Inc.), followed by brief vortexing. Non-targeting Silencer™ Negative Control No. 1 siRNA scramble negative transfection control RNA (Thermo Fisher Scientific, Inc.) and two different G6PDH siRNAs: G6PDH siRNA #1 (siRNA ID: S5448; Thermo Fisher Scientific, Inc.) and G6PDH siRNA #2 (siRNA ID: S531166; Thermo Fisher Scientific, Inc.) were diluted in Opti-MEM medium at a concentration of 2 pmol/10 µL (200 nM). Diluted Lipofectamine RNAiMAX and siRNAs were combined 1:1 ratio and distributed 10 µL per well (1 pmol final siRNA) in 100 µL of pre-warmed AEGM without antibiotics. The plate was incubated at 37 °C until immediately prior to Beas-2B seeding. After 48 h of transfection, Beas-2B cells were treated with B[a]P in fresh AEGM and handled analogously to non-transfected cells. Beas-2B cells were not re-transfected upon B[a]P treatment, as Western blot Analysis indicated incubation with fresh, siRNA-free AEGM kept G6PDH levels below 20% of scramble controls up to 48 h; data presented in Fig. 5b.

### Statistical analysis

Regression modeling and statistical analyses were carried out using R Program version 3.6.3. Statistical comparisons among groups were performed via two-tailed Student’s *t* test. One-sample *t* tests were performed on normalized or change from control endpoints; otherwise, two-sample *t* tests were performed with a significance below a probability of 0.05 considered significant. Heteroscedasticity and normality were assessed via the Levene’s test and the Anderson–Darling normality test, respectively.

Data presented are the arithmetic mean of independent experiments ± one standard error of the mean. Dose–response modeled was performed using the “drc” package in R, except for Fig. 1h, whose response deviated from regression analyses. Velocity of WST1 metabolism was also modeled using the “drc” package. Model selection based on Akaike Information Criteria. For clarity in endpoint-normalized co-incubation studies measuring cell cycle changes, WST1 metabolism, and EdU uptake, vehicle controls and inhibitor only controls were represented by a single bar denoted as a unified respective control group, denoted as “R. Ctrl”.

## Supplementary Information

Supplementary Information.

## Data Availability

The datasets generated during and/or analyzed during the current study are available from the corresponding author on reasonable request.
