# Supplementary material for: Variation in pentose phosphate pathway-associated metabolism dictates cytotoxicity outcomes determined by tetrazolium reduction assays
Source: Sci Rep. 2023 May 22;13:8220. doi: 10.1038/s41598-023-35310-5 (PMC10203251; doi:10.1038/s41598-023-35310-5)
Supplement: Supplementary file 1 — Supplementary Information. [file 41598_2023_35310_MOESM1_ESM.pdf]

Variation in Pentose Phosphate Pathway-associated Metabolism Dictates Cytotoxicity Outcomes Determined by Tetrazolium Reduction Assays

Jayme P. Coyle[1,4,*]

Caroline Johnson [1,2,4]

Jake Jensen[3]

Mariana Farcas[1,2]

Raymond Derk[1]

Todd A. Stueckle[1]

Tiffany G. Kornberg[1]

Yon Rojanasakul[2]

Liying W. Rojanasakul[1,4,*]

[1]HELD/ACIB, National Institute for Occupational Safety and Health, Morgantown, WV. [2]Department of Pharmaceutical Sciences, West Virginia University, Morgantown, WV. [3]Department of Environmental Health, Harvard University, Boston, MA. [4]J.P.C., C.J., and L.W.R. contributed equally to this work.

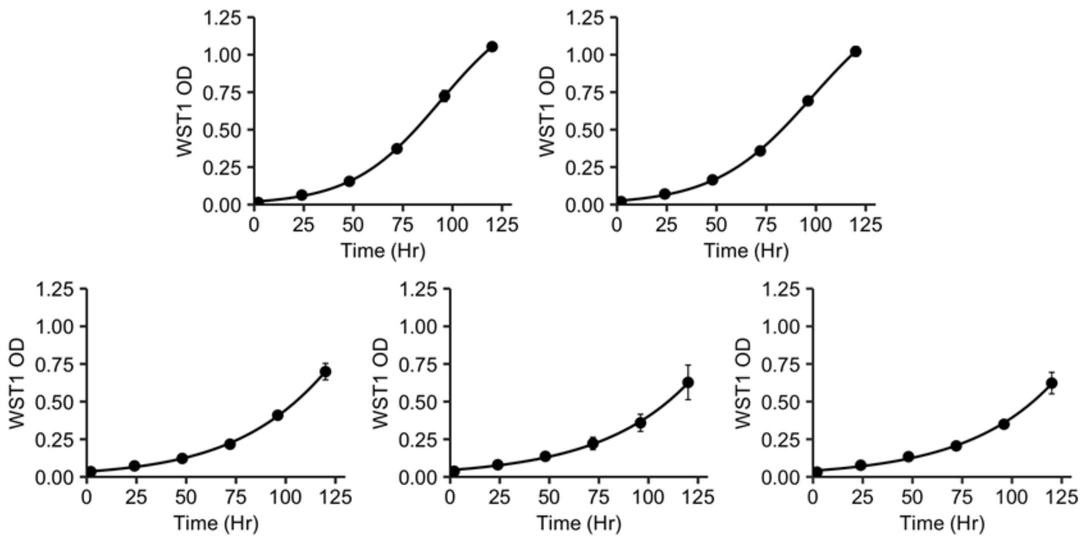

Supplementary Figure S1. Doubling Time WST1 Metabolism Curves. Beas-2B cells were treated with graded concentrations of B[a]P for 3 days, followed by doubling time assessment using WST. n = 3. Growth curves here were utilized for deriving doubling time (μmax^{-1}) shown in Figure 1K. Endpoints are the arithmetic mean listed independent experiments with error bars denoting one standard error of the mean.

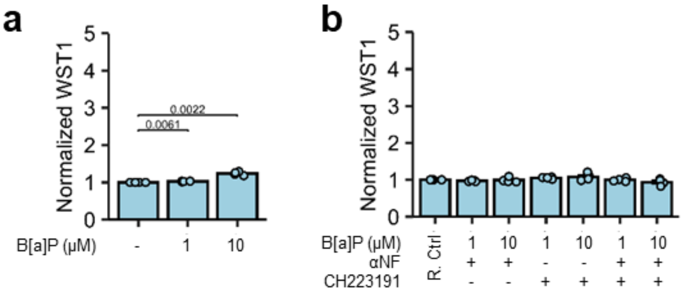

Supplementary Figure S2. B[a]P-induced WST1 Metabolic Changes 24 Hours Post-treatment. **a**, Beas-2B cells treated with B[a]P were assessed for WST1 metabolism. n = 5. **b**, Beas-2B cells treated with 1 μM or 10 μM B[a]P for 24 hours in the presence of 10 μM αNF (CYP and AhR inhibitor), 5 μM CH223191 (AhR Inhibitor), or both simultaneously, followed by WST1 metabolism. Respective vehicle- and inhibitor-only control (R. Ctrl). n = 3. Endpoints are the arithmetic mean of listed independent experiments with error bars denoting one standard error of the mean.

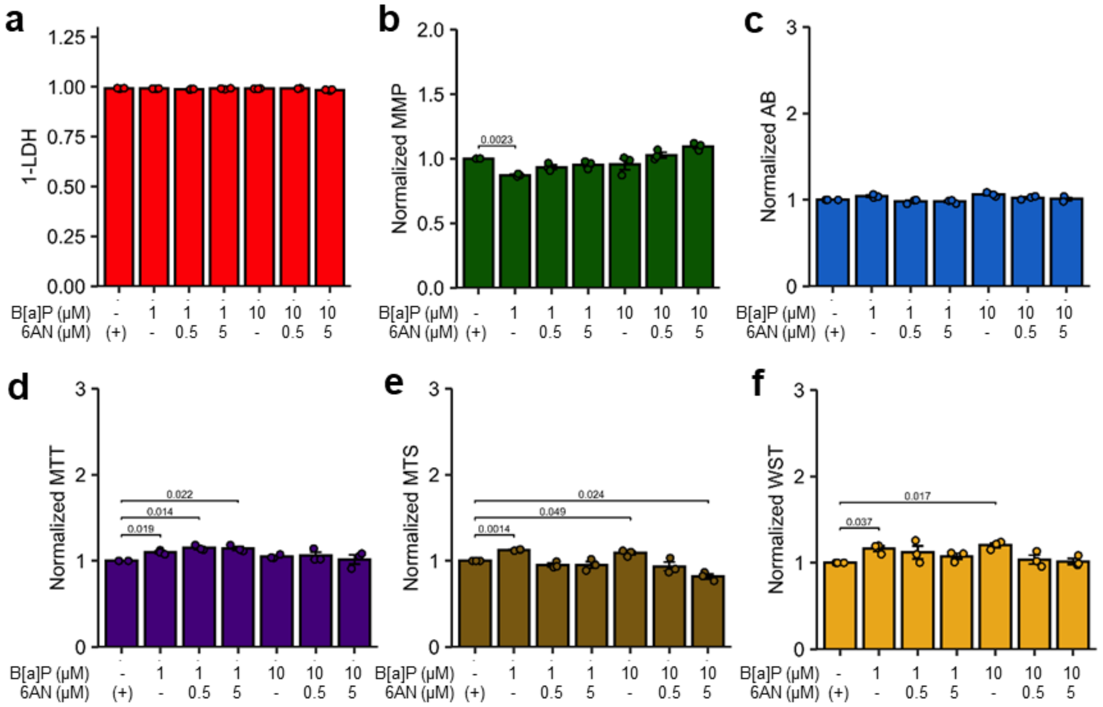

Supplementary Figure S3. Endpoint-specific Sensitivity to 6-Aminonicotinamide Co-treated with B[a]P**.** **a-f**, Beas-2B cells treated with graded concentrations of 6AN and B[a]P for 24 hours were assessed for **(a)** extracellular leakage via LDH and for **(b)** ΔΨm via JC-1, **(c)** Alamar Blue, **(d)** MTT, **(e)** MTS, and **(f)** WST1 metabolism. Vehicle- and inhibitor-only controls are represented by a unified group designated as R. Ctrl. n = 3. Endpoints are the arithmetic mean of listed independent experiments with error bars denoting one standard error of the mean.

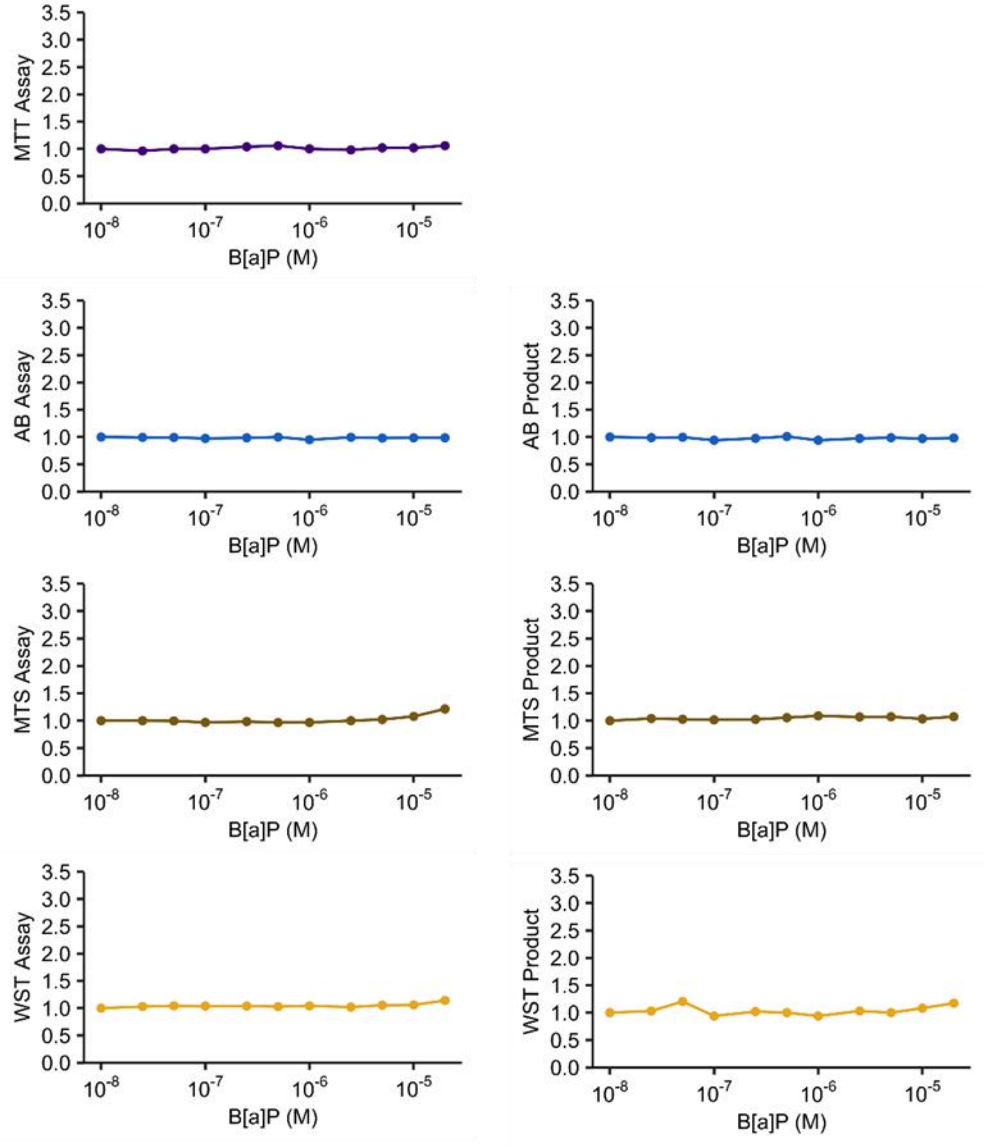

Supplementary Figure S4. Endpoint-specific Interference Assessment. Testing due to interference with assay performance [Left Column] and assay product [Right Column] for each specific resazurin and tetrazolium salt employed in this investigation. All point estimates are derived from a single experiment.

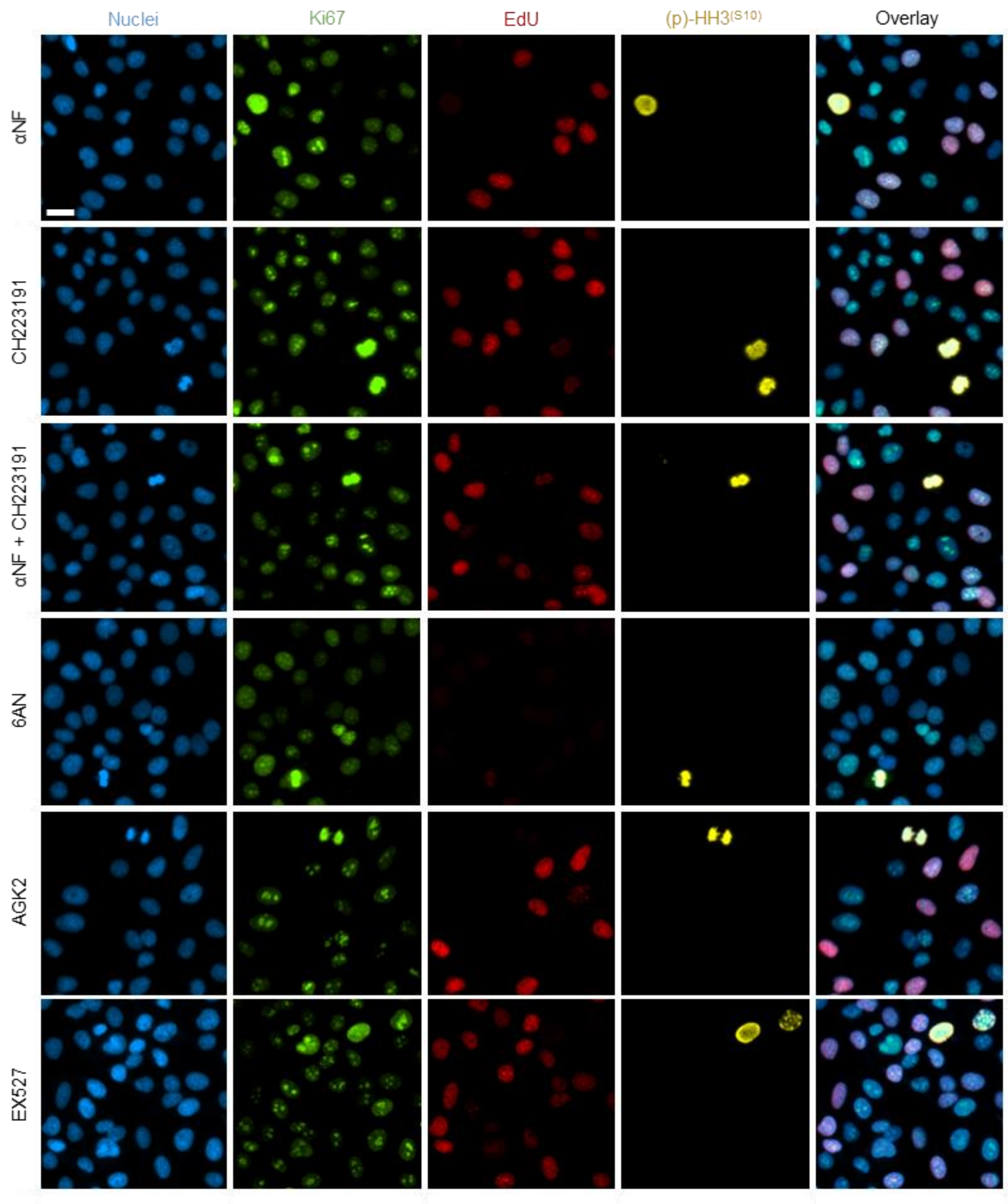

Supplementary Figure S5. Immunofluorescent Cell Cycle Assessment of Beas-2B Treated with Inhibitors. Inhibitor only-treated Beas-2B cells were fixed and stained for cell cycle analysis. Images were from experiments used for quantitative EdU and cell cycle analyses presented in **Fig. 3-4**. Images taken at 20X magnification, scale bar = 25 µm.

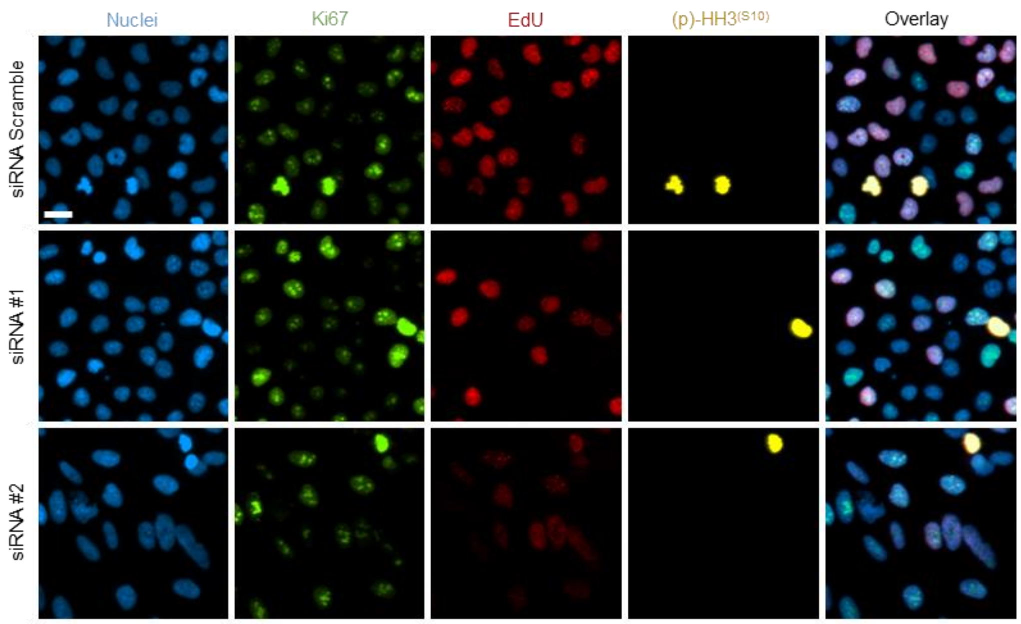

Supplementary Figure S6. Immunofluorescent Cell Cycle Assessment of Beas-2B Treated with siRNA. siRNA only-treated Beas-2B cells were fixed and stained for cell cycle analysis. Images were from experiments used for quantitative EdU and cell cycle analyses presented in **Fig. 5**. Images taken at 20X magnification, scale bar = 25 µm.

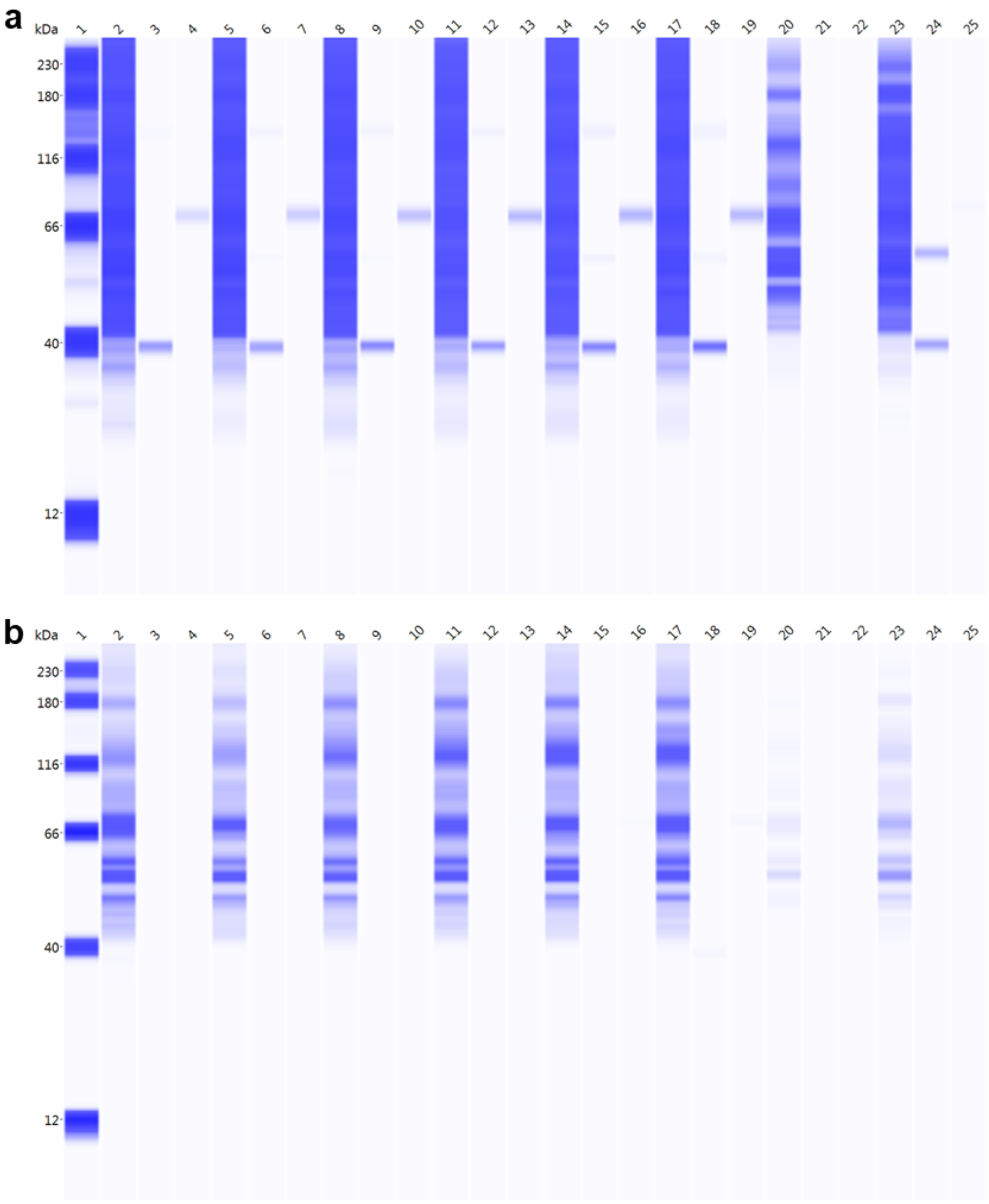

Supplementary Figure S7. Full Image Western Blot for control, B[a]P, nocodazole, and aphidicolin groups. **a.** Image for quantifying target proteins: (p)-cdc2(Y15), p)-Chk1(S345), and (p)-Chk2(T68). **b.** Image for quantifying respective total protein for normalizing target proteins. Lane annotation is shown in Supplementary Table S6.

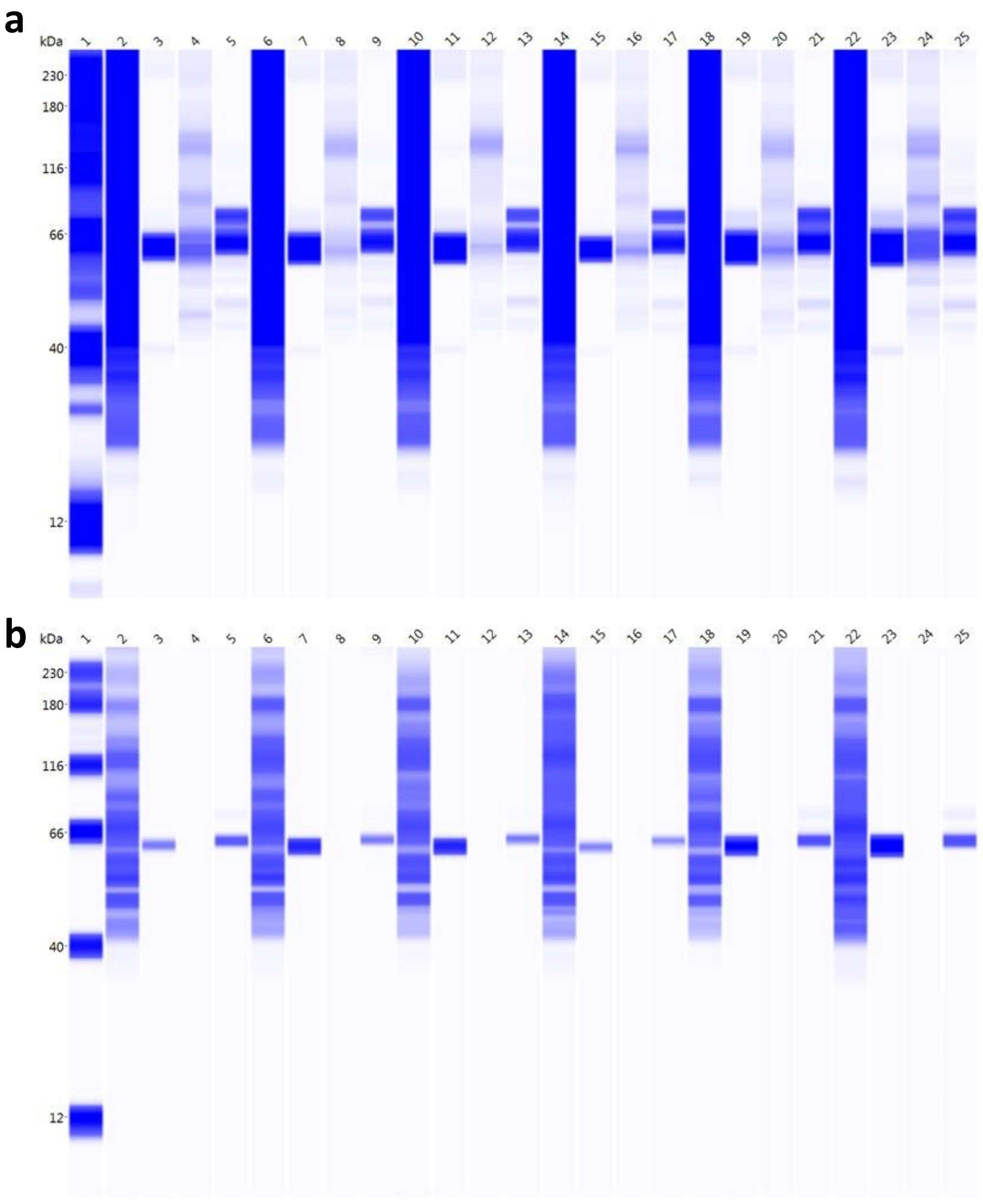

Supplementary Figure S8. Full Image Western Blot for control and B[a]P groups. **a.** Image for quantifying target proteins: (p)-cdc2(T161), p53 and (p)-p53(S15). **b.** Image for quantifying respective total protein for normalizing target proteins. Lane annotation is shown in Supplementary Table S7.

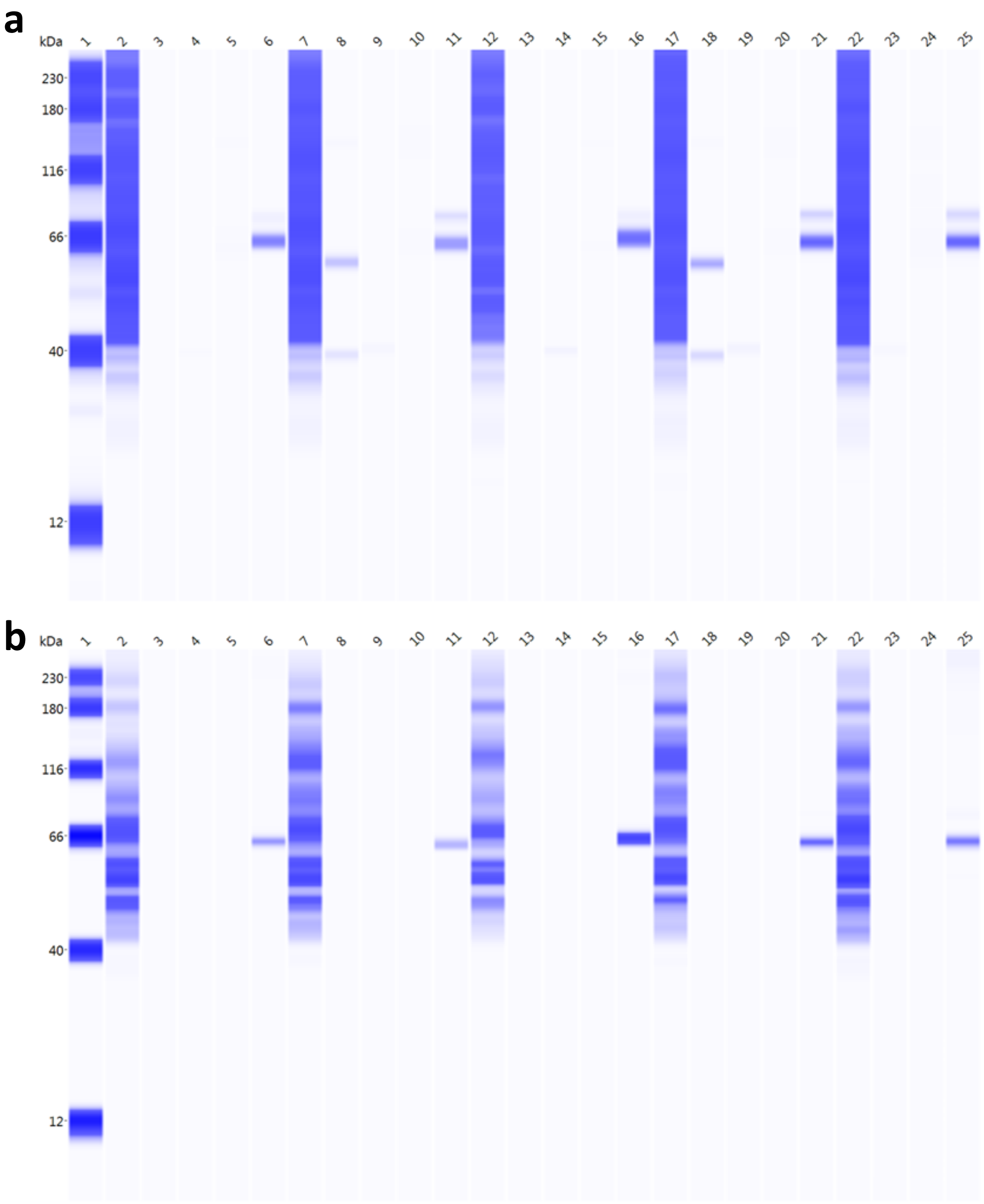

Supplementary Figure S9. Full Image Western Blot for nocodazole and aphidicolin groups. **a.** Image for quantifying target proteins: (p)-cdc2(T161), p)-cdc2(Y15), (p)-Chk1(S345), p-53, and (p)-p53(S15). **b.** Image for quantifying respective total protein for normalizing target proteins. Lane annotation is shown in Supplementary Table S8.

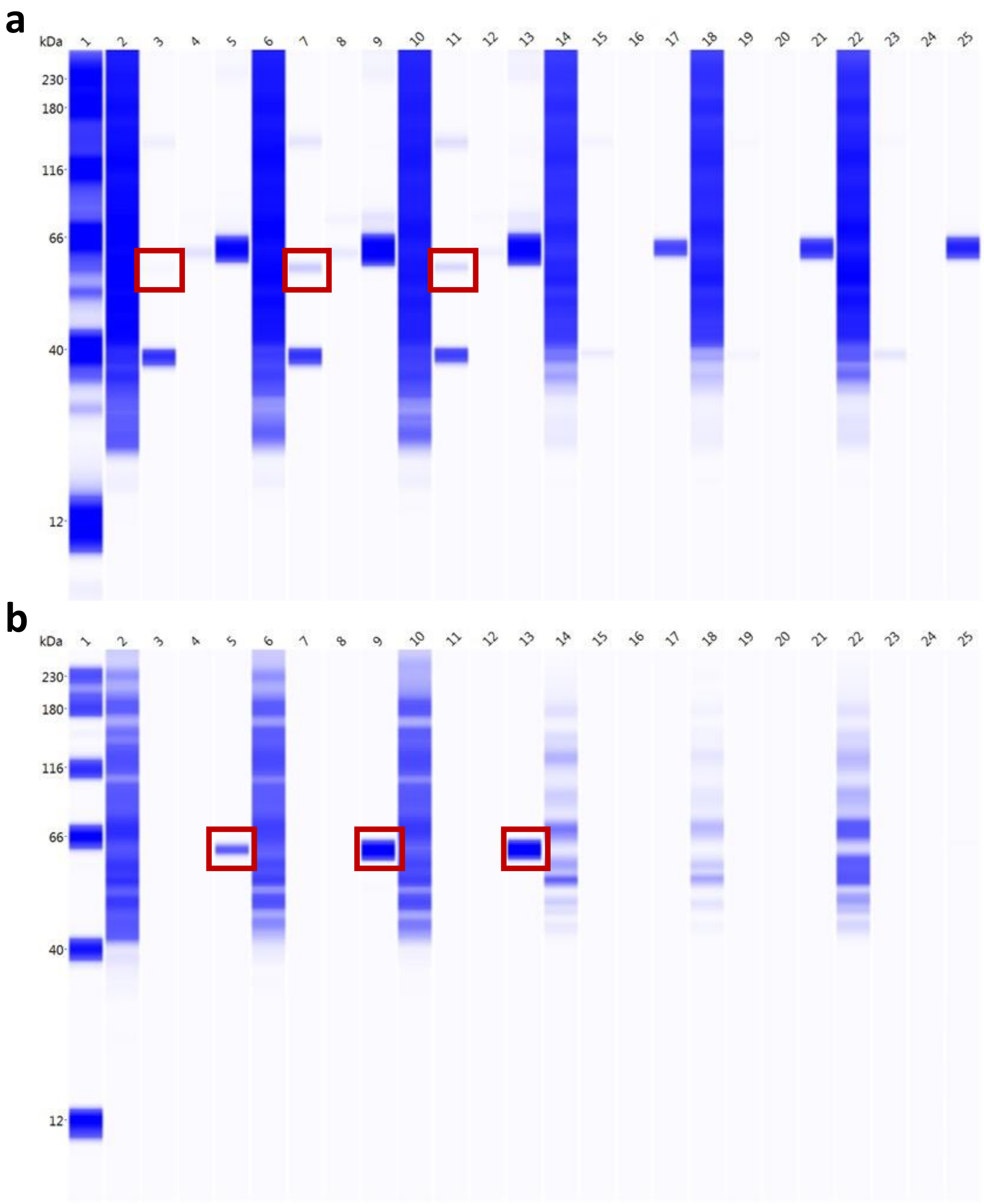

Supplementary Figure S10. Full Image Western Blot for control and B[a]P groups. **a.** Image for quantifying target proteins: (p)-cdc2(T161), p)-cdc2(Y15), (p)-Chk1(S345), p-53. **b.** Image for quantifying respective total protein for normalizing target proteins and (p)-p53(S15). Lane annotation is shown in Supplementary Table S9. Representative p-53 and (p)-p53(S15) bands presented in Figure 1 are outlined in red boxes.

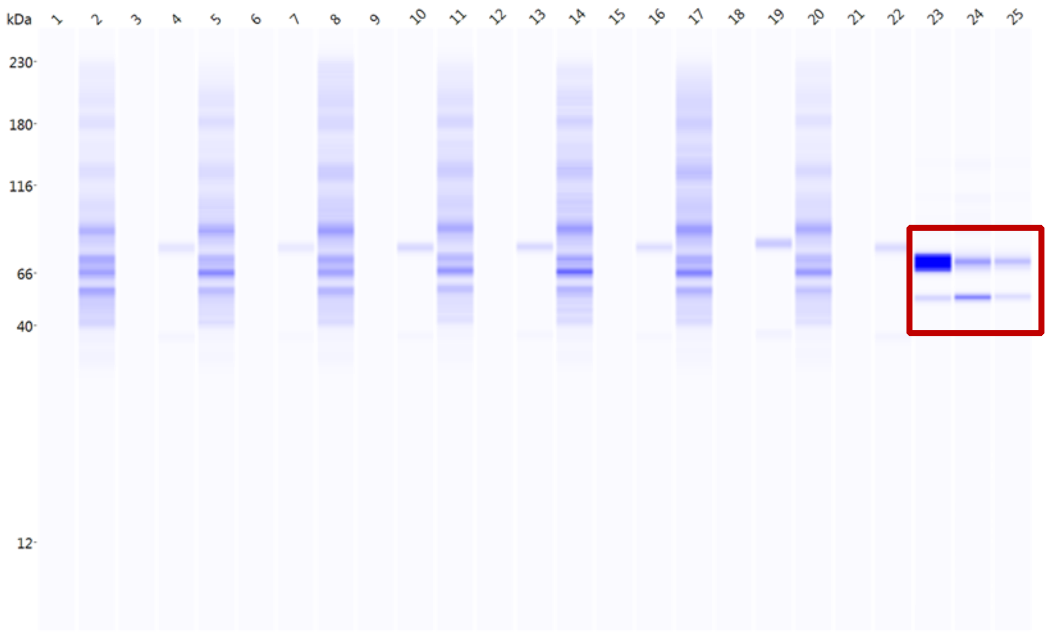

Supplementary Figure S11. Full Image Western Blot for control and B[a]P groups treated with G6PDH siRNA. Image for quantifying target proteins: G6PDH and β-actin. Lane annotation is shown in Supplementary Table S10. Representative bands presented in Figure 3 are outlined in red boxes.

Supplementary Table S1. Complete Tabulation of Cell Cycle Phase Prevalence

Treatment	Inhibitor	G_0	G_1	S	G_2	M
DMSO	None	1.7 ± 0.1	51.2 ± 0.6	36.0 ± 0.6	8.5 ± 0.2	2.7 ± 0.2
	αNF	2.8 ± 0.2	60.2 ± 1.0	24.6 ± 0.9	9.4 ± 0.3	3.0 ± 0.2
	CH223191	4.6 ± 0.7	49.3 ± 0.7	34.9 ± 0.9	7.8 ± 0.4	3.5 ± 0.3
	αNF + CH223191	2.5 ± 0.2	56.6 ± 2.7	29.5 ± 2.1	8.6 ± 0.4	2.9 ± 0.3
	AGK2	6.4 ± 1.0	48.3 ± 0.6	35.1 ± 0.8	7.6 ± 0.1	2.6 ± 0.1
	EX527	1.1 ± 0.1	48.7 ± 0.8	38.3 ± 0.9	8.7 ± 0.2	3.3 ± 0.3
	siRNA Scramble	5.6 ± 0.3	49.3 ± 0.5	33.5 ± 0.5	8.1 ± 0.2	3.5 ± 0.2
	siRNA #1	12.0 ± 0.4	57.7 ± 0.6	20.7 ± 0.2	6.9 ± 0.3	2.8 ± 0.1
	siRNA #2	9.3 ± 0.2	33.7 ± 0.6	30.6 ± 1.3	24.9 ± 1.4	1.5 ± 0.1
	6AN	1.4 ± 0.1	60.5 ± 1.6	23.9 ± 2.8	12.6 ± 1.0	1.7 ± 0.2
1 μM B[a]P	None	1.5 ± 0.1	42.0 ± 0.5	45.8 ± 0.7	8.3 ± 0.2	2.3 ± 0.1
	αNF	3.3 ± 0.4	58.0 ± 0.2	25.6 ± 0.6	10.3 ± 0.2	2.8 ± 0.2
	CH223191	2.9 ± 0.2	44.3 ± 0.3	42.6 ± 0.6	7.9 ± 0.4	2.4 ± 0.2
	αNF + CH223191	2.3 ± 0.3	57.4 ± 2.5	29.3 ± 2.2	8.5 ± 0.5	2.6 ± 0.2
	AGK2	6.7 ± 1.4	47.1 ± 0.3	37.1 ± 1.3	7.2 ± 0.2	2.0 ± 0.1
	EX527	0.8 ± 0.2	42.0 ± 0.5	48.0 ± 1.1	7.2 ± 0.2	2.0 ± 0.3
	siRNA Scramble	8.0 ± 0.3	36.0 ± 0.5	45.9 ± 0.1	8.7 ± 0.4	1.5 ± 0.0
	siRNA #1	11.8 ± 0.7	43.3 ± 1.7	25.8 ± 0.3	17.4 ± 0.9	1.6 ± 0.1
	siRNA #2	7.9 ± 0.5	35.0 ± 1.0	25.1 ± 3.1	31.2 ± 1.5	0.8 ± 0.1
	6AN	1.6 ± 0.2	63.4 ± 1.9	17.3 ± 3.1	15.7 ± 0.9	1.9 ± 0.2
10 μM B[a]P	None	2.8 ± 0.3	41.6 ± 0.6	44.9 ± 0.6	8.7 ± 0.1	2.0 ± 0.1
	αNF	3.4 ± 0.4	60.0 ± 0.9	24.7 ± 1.0	9.7 ± 0.2	2.3 ± 0.2
	CH223191	3.3 ± 0.4	44.7 ± 0.1	41.6 ± 1.1	8.4 ± 0.6	2.1 ± 0.1
	αNF + CH223191	2.8 ± 0.4	54.5 ± 1.3	31.1 ± 1.1	8.9 ± 0.5	2.7 ± 0.2
	AGK2	6.4 ± 1.1	43.7 ± 0.5	40.5 ± 1.5	7.4 ± 0.1	1.9 ± 0.0
	EX527	0.9 ± 0.0	42.3 ± 0.3	47.3 ± 0.4	7.4 ± 0.1	2.1 ± 0.2
	siRNA Scramble	11.2 ± 0.4	38.6 ± 0.8	38.4 ± 0.4	9.8 ± 0.2	2.1 ± 0.1
	siRNA #1	14.5 ± 0.8	40.8 ± 1.6	24.8 ± 0.0	18.2 ± 0.8	1.7 ± 0.1
	siRNA #2	8.6 ± 0.4	32.4 ± 1.0	29.1 ± 3.0	29.0 ± 1.7	0.8 ± 0.0
	6AN	2.1 ± 0.3	66.9 ± 1.8	11.5 ± 2.4	17.8 ± 0.4	1.7 ± 0.1
Aphidicolin	None	0.8 ± 0.1	69.9 ± 2.9	0.7 ± 0.2	28.7 ± 2.8	0.0 ± 0.0
Nocodazole	None	10.8 ± 0.6	6.4 ± 0.1	2.5 ± 0.6	6.0 ± 1.2	74.2 ± 1.3

All values are the arithmetic mean of 3 independent experiments ± one standard error of the mean, except Nocodazole-treated Beas-2B cells. Values for this group are the arithmetic mean of 2 independent experiments ± one standard error of the mean

Supplementary Table S2. Inhibitors Employed

Inhibitor	Vendor	Catalog<br>Lot	Solvent	[M]
Aphidicolin	Sigma	A0781<br>077M4009V	DMSO	3 µg/mL
Nocodazole	Sigma	M1404<br>038M4007V	DMSO	0.1 µg/mL
6AN	Sigma	A68203<br>SHBH5345V	DMSO	10 µM
αNF	Sigma	N5757<br>SLBX8269	DMSO	10 µM
CH223191	Sigma	C8124<br>0000042799	DMSO	5 µM
AGK2	Caymen<br>Chemical	13145<br>0506253-14	DMSO	10 µM
EX527	APExBIO	A4181<br>2	DMSO	0.5 µM

Supplementary Table S3. High-Content Imaging Platform Specifications

ImageXpress Micro XLS<br>Serial 122603	
Camera and Light Source	
Light Source	Solid State LED<br>380-650 nm Range
Camera	4.66 MP Camera<br>CMOS Detector
Objectives	
10X	Nikon Plan Fluor<br>0.3 NA
20X	Nikon Ph1 S Plan Fluor<br>ELWD ADM 0.45 NA

Supplementary Table S4. High-Content Imaging Platform Filter Cubes

Cube	Excitation Wavelength	Emission Wavelength	Dichroic Mirror	Fluorophore
DAPI	377/50	447/60	327-404 (R)<br>415-950 (T)	Hoechst 33342
FITC	482/35	536/40	350-500 (R)<br>513-950 (T)	Alexa-488
TRITC	543/22	593/40	350-555 (R)<br>569-950 (T)	Alexa-555
Cy5	628/40	692/40	350-651 (R)<br>669-950(T)	Alexa-647

Supplementary Table S5. Imaging Parameters

Method	Channel	Exposure Time (ms)	Imaging Depth	Imaging Medium
Enhanced Cell Cycle	DAPI<br>FITC<br>TRITC<br>Cy5	18<br>80<br>40<br>800	16-bit<br>16-bit<br>16-bit<br>16-bit	DPBS<br>22°C
Live Cell Imaging	DAPI<br>Cy5	18<br>600	16-bit<br>14-bit	AEGM

*Imaging bit depth

Supplementary Table S6. Lane Annotation for Supplementary Figure S7.

Lane	***Sample***	***Target 1***	***Target 2***
1	Ladder	Ladder	
2	(-) Control 24 Hr	Total Protein (Normalization)	
3	(-) Control 24 Hr	(p)-cdc2(Y15)	(p)-Chk1(S345)
4	(-) Control 24 Hr	(p)-Chk2(T68)	
5	1 µM B[a]P 24 Hr	Total Protein (Normalization)	
6	1 µM B[a]P 24 Hr	(p)-cdc2(Y15)	(p)-Chk1(S345)
7	1 µM B[a]P 24 Hr	(p)-Chk2(T68)	
8	10 µM B[a]P 24 Hr	Total Protein (Normalization)	
9	10 µM B[a]P 24 Hr	(p)-cdc2(Y15)	(p)-Chk1(S345)
10	10 µM B[a]P 24 Hr	(p)-Chk2(T68)	
11	(-) Control 24 Hr	Total Protein (Normalization)	
12	(-) Control 24 Hr	(p)-cdc2(Y15)	(p)-Chk1(S345)
13	(-) Control 24 Hr	(p)-Chk2(T68)	
14	1 µM B[a]P 24 Hr	Total Protein (Normalization)	
15	1 µM B[a]P 24 Hr	(p)-cdc2(Y15)	(p)-Chk1(S345)
16	1 µM B[a]P 24 Hr	(p)-Chk2(T68)	
17	10 µM B[a]P 24 Hr	Total Protein (Normalization)	
18	10 µM B[a]P 24 Hr	(p)-cdc2(Y15)	(p)-Chk1(S345)
19	10 µM B[a]P 24 Hr	(p)-Chk2(T68)	
20	Nocodazole 18 Hr	Total Protein (Normalization)	
21	Nocodazole 18 Hr	(p)-cdc2(Y15)	(p)-Chk1(S345)
22	Nocodazole 18 Hr	(p)-Chk2(T68)	
23	Aphidicolin 18 Hr	Total Protein (Normalization)	
24	Aphidicolin 18 Hr	(p)-cdc2(Y15)	(p)-Chk1(S345)
25	Aphidicolin 18 Hr	(p)-Chk2(T68)	

Supplementary Table S7. Lane Annotation for Supplementary Figure S8.

Lane	***Sample***	***Target 1***	***Target 2***
1	Ladder	Ladder	
2	(-) Control 24 Hr	Total Protein (Normalization)	
3	(-) Control 24 Hr	(p)-cdc2(T161)	(p)-p53(S15)
4	(-) Control 24 Hr	p53	
5	(-) Control 24 Hr	Empty	
6	1 µM B[a]P 24 Hr	Total Protein (Normalization)	
7	1 µM B[a]P 24 Hr	(p)-cdc2(T161)	(p)-p53(S15)
8	1 µM B[a]P 24 Hr	p53	
9	1 µM B[a]P 24 Hr	Empty	
10	10 µM B[a]P 24 Hr	Total Protein (Normalization)	
11	10 µM B[a]P 24 Hr	(p)-cdc2(T161)	(p)-p53(S15)
12	10 µM B[a]P 24 Hr	p53	
13	10 µM B[a]P 24 Hr	Empty	
14	(-) Control 24 Hr	Total Protein (Normalization)	
15	(-) Control 24 Hr	(p)-cdc2(T161)	(p)-p53(S15)
16	(-) Control 24 Hr	p53	
17	(-) Control 24 Hr	Empty	
18	1 µM B[a]P 24 Hr	Total Protein (Normalization)	
19	1 µM B[a]P 24 Hr	(p)-cdc2(T161)	(p)-p53(S15)
20	1 µM B[a]P 24 Hr	p53	
21	1 µM B[a]P 24 Hr	Empty	
22	10 µM B[a]P 24 Hr	Total Protein (Normalization)	
23	10 µM B[a]P 24 Hr	(p)-cdc2(T161)	(p)-p53(S15)
24	10 µM B[a]P 24 Hr	p53	
25	10 µM B[a]P 24 Hr	Empty	

Supplementary Table S8. Lane Annotation for Supplementary Figure S9.

Lane	***Sample***	***Target 1***	***Target 2***
1	Ladder	Ladder	
2	Nocodazole 18 Hr	Total Protein (Normalization)	
3	Nocodazole 18 Hr	(p)-cdc2(Y15)	(p)-Chk1(S345)
4	Nocodazole 18 Hr	(p)-cdc2(T161)	(p)-p53(S15)
5	Nocodazole 18 Hr	p53	
6	Nocodazole 18 Hr	(p)-Chk2(T68)	
7	Aphidicolin 18 Hr	Total Protein (Normalization)	
8	Aphidicolin 18 Hr	(p)-cdc2(Y15)	(p)-Chk1(S345)
9	Aphidicolin 18 Hr	(p)-cdc2(T161)	(p)-p53(S15)
10	Aphidicolin 18 Hr	p53	
11	Aphidicolin 18 Hr	(p)-Chk2(T68)	
12	Nocodazole 18 Hr	Total Protein (Normalization)	
13	Nocodazole 18 Hr	(p)-cdc2(Y15)	(p)-Chk1(S345)
14	Nocodazole 18 Hr	(p)-cdc2(T161)	(p)-p53(S15)
15	Nocodazole 18 Hr	p53	
16	Nocodazole 18 Hr	(p)-Chk2(T68)	
17	Aphidicolin 18 Hr	Total Protein (Normalization)	
18	Aphidicolin 18 Hr	(p)-cdc2(Y15)	(p)-Chk1(S345)
19	Aphidicolin 18 Hr	(p)-cdc2(T161)	(p)-p53(S15)
20	Aphidicolin 18 Hr	p53	
21	Aphidicolin 18 Hr	(p)-Chk2(T68)	
22	Aphidicolin 18 Hr	Total Protein (Normalization)	
23	Aphidicolin 18 Hr	(p)-cdc2(Y15)	(p)-Chk1(S345)
24	Aphidicolin 18 Hr	(p)-cdc2(T161)	(p)-p53(S15)
25	Aphidicolin 18 Hr	p53	

Supplementary Table S9. Lane Annotation for Supplementary Figure S10.

Lane	*Sample*	*Target 1*	*Target 2*	*Note*
1	Ladder	Ladder		
2	(-) Control 24 Hr	Total Protein (Normalization)		
3	(-) Control 24 Hr	(p)-cdc2(Y15)	(p)-Chk1(S345)	
4	(-) Control 24 Hr	(p)-Chk2(T68)	p53	Figure 1j
5	(-) Control 24 Hr	(p)-p53(S15)		Figure 1j
6	1 µM B[a]P 24 Hr	Total Protein (Normalization)		
7	1 µM B[a]P 24 Hr	(p)-cdc2(Y15)	(p)-Chk1(S345)	
8	1 µM B[a]P 24 Hr	(p)-Chk2(T68)	p53	Figure 1j
9	1 µM B[a]P 24 Hr	(p)-p53(S15)		Figure 1j
10	10 µM B[a]P 24 Hr	Total Protein (Normalization)		
11	10 µM B[a]P 24 Hr	(p)-cdc2(Y15)	(p)-Chk1(S345)	
12	10 µM B[a]P 24 Hr	(p)-Chk2(T68)	p53	Figure 1j
13	10 µM B[a]P 24 Hr	(p)-p53(S15)		Figure 1j
14	(-) Control 24 Hr	Total Protein (Normalization)		
15	(-) Control 24 Hr	(p)-cdc2(Y15)	(p)-Chk1(S345)	
16	(-) Control 24 Hr	(p)-Chk2(T68)	p53	
17	(-) Control 24 Hr	(p)-p53(S15)		
18	1 µM B[a]P 24 Hr	Total Protein (Normalization)		
19	1 µM B[a]P 24 Hr	(p)-cdc2(Y15)	(p)-Chk1(S345)	
20	1 µM B[a]P 24 Hr	(p)-Chk2(T68)	p53	
21	1 µM B[a]P 24 Hr	(p)-p53(S15)		
22	10 µM B[a]P 24 Hr	Total Protein (Normalization)		
23	10 µM B[a]P 24 Hr	(p)-cdc2(Y15)	(p)-Chk1(S345)	
24	10 µM B[a]P 24 Hr	(p)-Chk2(T68)	p53	
25	10 µM B[a]P 24 Hr	(p)-p53(S15)		

Note designates in which figure the lane has been presented as a representative electrophoresis

Supplementary Table S10. Lane Annotation for Supplementary Figure S11.

Lane	*Sample*	*Target 1*	*Target 2*	*Note*
1	Redacted	Redacted	Redacted	
2	Redacted	Redacted	Redacted	
3	Redacted	Redacted	Redacted	
4	Redacted	Redacted	Redacted	
5	Redacted	Redacted	Redacted	
6	Redacted	Redacted	Redacted	
7	Redacted	Redacted	Redacted	
8	Redacted	Redacted	Redacted	
9	Redacted	Redacted	Redacted	
10	Redacted	Redacted	Redacted	
11	Redacted	Redacted	Redacted	
12	Redacted	Redacted	Redacted	
13	Redacted	Redacted	Redacted	
14	Redacted	Redacted	Redacted	
15	Redacted	Redacted	Redacted	
16	Redacted	Redacted	Redacted	
17	Redacted	Redacted	Redacted	
18	Redacted	Redacted	Redacted	
19	Redacted	Redacted	Redacted	
20	Redacted	Redacted	Redacted	
21	Redacted	Redacted	Redacted	
22	Redacted	Redacted	Redacted	
23	(-) Control - Scr. SiRNA	G6PDH	B-Actin	Figure 3f
24	(-) Control - SiRNA #1	G6PDH	B-Actin	Figure 3f
25	(-) Control - SiRNA #2	G6PDH	B-Actin	Figure 3f

Note designates in which figure the lane has been presented as a representative electrophoresis.
Redacted indicates samples were not affiliated with the current investigation.
